# Pretransition state and apo structures of the filament-forming enzyme SgrAI elucidate mechanisms of activation and substrate specificity

**DOI:** 10.1016/j.jbc.2022.101760

**Published:** 2022-02-21

**Authors:** Zelin Shan, Niloofar Ghadirian, Dmitry Lyumkis, Nancy.C. Horton

**Affiliations:** 1 Laboratory of Genetics, The Salk Institute of Biological Sciences, La Jolla, California, USA; 2 Department of Chemistry and Biochemistry, University of Arizona, Tucson, Arizona, USA; 3 Department of Integrative Structural and Computational Biology, The Scripps Research Institute, La Jolla, California, USA; 4 Department of Molecular and Cellular Biology, University of Arizona, Tucson, Arizona, USA

**Keywords:** filament-forming enzymes, allostery, enzyme catalysis, substrate specificity modulation, order-to-disorder transitions, CTF, contrast transfer function, DBD, DNA-bound SgrAI dimer, FSC, Fourier shell correlation, PDB, Protein Data Bank, ROO, run-on oligomeric, SP, scissile phosphate or scissile phosphodiester group, UCSF, University of California San Francisco

## Abstract

Enzyme filamentation is a widespread phenomenon that mediates enzyme regulation and function. For the filament-forming sequence-specific DNA endonuclease SgrAI, the process of filamentation both accelerates its DNA cleavage activity and expands its DNA sequence specificity, thus allowing for many additional DNA sequences to be rapidly cleaved. Both outcomes—the acceleration of DNA cleavage and the expansion of sequence specificity—are proposed to regulate critical processes in bacterial innate immunity. However, the mechanistic bases underlying these events remain unclear. Herein, we describe two new structures of the SgrAI enzyme that shed light on its catalytic function. First, we present the cryo-EM structure of filamentous SgrAI bound to intact primary site DNA and Ca^2+^ resolved to ∼2.5 Å within the catalytic center, which represents the trapped enzyme–DNA complex prior to the DNA cleavage reaction. This structure reveals important conformational changes that contribute to the catalytic mechanism and the binding of a second divalent cation in the enzyme active site, which is expected to contribute to increased DNA cleavage activity of SgrAI in the filamentous state. Second, we present an X-ray crystal structure of DNA-free (apo) SgrAI resolved to 2.0 Å resolution, which reveals a disordered loop involved in DNA recognition. Collectively, these multiple new observations clarify the mechanism of expansion of DNA sequence specificity of SgrAI, including the indirect readout of sequence-dependent DNA structure, changes in protein–DNA interactions, and the disorder-to-order transition of a crucial DNA recognition element.

Filamentation of enzymes involving their end-to-end polymerization plays critical and diverse roles in many biological pathways, including metabolism (carbohydrate, amino acid, fatty acid, and nucleic acid), translation, innate immunity, scaffolding, and intracellular signaling, among many others (1). Despite being known for many decades, enzyme filamentation has only recently become appreciated as a widespread and evolutionarily conserved phenomenon (1, 2, 3, 4). However, the role of filamentation in regulating enzyme function is much less well understood and likely varies with the enzyme in question. Some possible roles include the alteration of enzyme specificity, activation or inhibition of enzyme activity, control of enzyme localization, nucleation, and stabilization of phase-separated compartments in cells, or even modulation of cellular shape (1, 5). Many of the same enzymes that form filaments composed of polymerized enzymes also form large self-assemblies in cells (sometimes also referred to as “filaments,” “cytoophidia,” or “rods and rings” in the literature) (1, 6, 7, 8, 9, 10, 11). The function of the self-assemblies, and the relationship between enzyme polymeric filaments and cellular self-assemblies, has yet to be elucidated in most cases (1).

We study type II restriction endonuclease SgrAI to better understand the molecular mechanism of enzyme filamentation in the control of enzyme activity and to define the biological advantages filamentation provides over other forms of enzyme regulation. Our discovery that SgrAI forms filaments was completely unexpected, and our work has since shown that filamentation leads to altered enzymatic properties in both the rate of DNA cleavage by SgrAI and in its substrate specificity (*i.e.*, the sequences of DNA cleaved by the enzyme) (12, 13, 14). Under basal conditions, SgrAI is a homodimeric and sequence-specific restriction endonuclease of 74 kDa with two active sites, one in each subunit. Each active site cleaves bound duplex DNA containing any one of 17 different 8 bp recognition sites. Three of these sites, known as primary sequences or primary sites, have the pattern CRCCGGYG (where R = A or G and Y = C or T) and can be cleaved by SgrAI in the absence of filamentation (12, 15). The 14 additional double-stranded sequences cleaved by SgrAI, known as secondary sequences or secondary sites, differ from primary sites by the substitution of a single bp in either the first or second position; these contain the patterns CCCCGGYG and DRCCGGYG, where D = A, T, or G. Secondary site sequences are cleaved appreciably only in the presence of sufficient concentrations of SgrAI bound to primary site containing DNA and under filament-forming conditions.

Analytical ultracentrifugation and native gel electrophoresis studies of SgrAI revealed hypermultimerization of DNA-bound SgrAI dimers (DBDs) under conditions wherein DNA cleavage activity is enhanced by several orders of magnitude, resulting in cleavage of both primary and secondary site sequences (12). Data from ion mobility native mass spectrometry not only confirmed the heterogeneous nature of SgrAI–DNA assemblies, but also indicated that they exhibited a regular repeating structure, which is suggestive of a filament (13). EM was instrumental in demonstrating the filamentous nature of SgrAI bound to its primary site sequence and revealed that DNA-bound SgrAI assembles into filaments in a run-on manner, characterized by the addition of individual DBDs at either end (14). Additional biophysical and DNA cleavage studies indicated that SgrAI bound to secondary sequences cannot alone induce filamentation but will assemble into filaments formed by SgrAI bound to primary site DNA (12). Since DNA cleavage activity is greatly accelerated in the filamentous form, we proposed that interactions between DBDs within the filament stabilize an activated conformation of the enzyme that is distinct from that exhibited in the nonfilamentous state (12, 14, 16). Cleavage of secondary sequences under activating conditions is explained by our model in that DBDs containing secondary sequences will be drawn into filaments composed of DBDs containing primary sequences, which activates the filamentous assembly to rapidly cleave bound secondary site DNAs (12, 17).

To better understand this regulatory mechanism, we previously determined structures of SgrAI bound to both primary and secondary sequences and in both filamentous and nonfilamentous states. These structures showed how the primary sequence is recognized by SgrAI, using both direct and indirect readout of the DNA sequence, and provided initial insight into the mechanism of DNA cleavage (14, 16, 18, 19). For Mg^2+^-dependent DNA nucleases, such as SgrAI, two Mg^2+^ ions are typically expected to bind per enzyme active site in what is known as the two-metal ion mechanism (20, 21, 22). These ions bridge the protein–DNA interface at the site of phosphodiester cleavage and perform critical roles in catalysis, such as activation of the nucleophile (a metal ion–bound water molecule), stabilization of the transition state, and stabilization of the leaving group of the reaction (20, 21, 22, 23). Interestingly, crystal structures of nonfilamentous low activity SgrAI bound to primary site DNA show that only one of the two important metal ions resides in a location predicted by the two-metal ion mechanism, whereas a second ion is located ∼4 to 5 Å from its expected position (18). We proposed that this “mispositioning” of one of two Mg^2+^ ions accounts for the slow DNA cleavage rate of SgrAI in the nonfilamentous state, and that enzyme activation occurs *via* a conformational change of the SgrAI–DNA complex stabilized within the filament, which results in tight Mg^2+^ binding to the second canonical site (18, 19).

To investigate hypotheses regarding the origin of activation of SgrAI, we previously determined multiple structures of filamentous SgrAI bound to primary site DNA using cryo-EM at 8.6 Å and later at 3.5 Å global resolution (14, 16). The structures revealed a left-handed helical filament with approximately four DBDs per turn and a large conformational change consisting of a rotation between subunits of approximately 11° in comparison to the nonfilamentous conformation (14). We found extensive interactions between neighboring DBDs in the filament, which could not occur without the change in conformation (14). The 8.6 Å resolution of the first reported structure was, however, insufficient to reveal details of the conformational change; the higher resolution structure at ∼3.5 Å allowed for visualization of significant shifts in amino acid positions at the dimeric interface of the enzyme, which result directly from the intersubunit rotation (16). One protein segment (residues Gly181–Asp188) extends from the dimeric interface to the protein–DNA interface within the enzymatic active site, where DNA cleavage is catalyzed. We proposed that this shift created a tighter binding site for the second metal ion predicted by the two-metal ion mechanism but that was absent in the structures of the low-activity nonfilamentous conformation. However, an important limitation of this structure was that the synthetic DNA used for structure determination did not contain the scissile phosphate or scissile phosphodiester (SP) group that is cleaved by SgrAI. We previously proposed that the absence of the SP was responsible for the lack of occupancy of the second metal ion–binding site and that structures with contiguous DNA containing this important chemical group would support its occupancy and thereby reveal novel insights into the mechanism of enzymatic activity (16).

The filamentous structures also provided insight into the molecular mechanism of secondary site cleavage specificity by SgrAI (14, 16). When compared with the structure of SgrAI bound to either primary or secondary site DNA in the nonfilamentous form, the earlier 3.5 Å resolution structure of filamentous SgrAI indicated a change in the base stacking of the bound DNA at the base pair substituted in a subset of secondary sequences (*i.e.*, those substituted in the second position, CCCCGGYG). From this observation, we proposed a mechanism for secondary site activity resulting from changes in the energy landscape of low-activity and high-activity conformations of SgrAI, which stem from changes in base stacking energies. Specifically, our model proposes that the conformation of DNA in the low-activity nonfilamentous form is more preferred when secondary site sequences are bound than that of the high-activity filamentous form, which as a result decreases the propensity of SgrAI to filament when bound to secondary site sequences substituted at the second bp (16). However, the prior structures did not provide any insight into how secondary site sequences containing substitutions in the first bp (*i.e.*, DRCCGGYG) influenced SgrAI behavior.

There are numerous outstanding questions pertaining to SgrAI structure, function, and the intriguing ways in which its activity and specificity are modulated through filamentation. How many metal ions are present in the active site in the activated enzyme conformation? What is their role during different steps of the enzymatic cleavage reaction? What is the origin of primary and secondary site cleavage specificities, and how do these influence the propensity of SgrAIs to form higher-order assemblies? To address these questions, it is necessary to capture the enzyme in distinct states and use high-resolution structural biology to piece together a mechanistic understanding of enzyme function. Herein, we present two novel structures of the SgrAI enzyme, in the filamentous state bound to intact primary site DNA containing the SP, and in the apo state without DNA. The findings reveal numerous novel insights into the mechanism of activity of SgrAI and the enzyme's allosteric modulation of DNA cleavage specificity.

## Results

### Filament assembly and structure determination of activated SgrAI bound to DNA containing the SP

We set out to determine the structure of SgrAI bound to DNA containing the full phosphodiester bond at the cleavage site. We used Ca^2+^ in place of the biologically relevant and catalytically competent metal ion Mg^2+^ to capture the active site prior to the DNA cleavage. Ca^2+^ ions often bind in the same or similar locations within the active site of DNA nucleases, but the use of these ions has the advantage of stalling DNA cleavage to provide a view of the active site prior to commencement of the reaction (24, 25). The full phosphodiester moiety at the site of cleavage is important, since arrangements of divalent cations and other active site groups can be influenced by its presence.

We prepared SgrAI run-on oligomeric (ROO) filaments from purified, recombinant, and wildtype protein bound to a 40 bp oligonucleotide DNA containing the primary site sequence CACCGGTG using methods previously described (16). Filaments were vitrified on cryo-EM grids, and we collected a total of 1047 movies containing SgrAI filaments. The filaments were heterogeneous in length because of the run-on oligomerization occurring during the process of filament formation but typically were limited to <10 DBDs. We used template-based particle matching to select filaments and processed the data in a conventional single-particle manner, with the exception that helical symmetry was imposed during the final reconstruction. This procedure, previously described for the lower-resolution ROO filament without the SP (16), produced a map resolved to ∼2.7 Å resolution globally, with regions of the map within the central portion of the filament resolved to ∼2.5 Å locally (Fig. S1 and Table S1). Notably, the active site of the enzyme is located within the central portion of the filament, and therefore, this important region was one of the best resolved regions of the map. We then derived a model of the SgrAI enzyme in the filamentous form. Because of the high resolution of the reconstructed map, the model contained metal ions and many solvent molecules that could be confidently modeled, including within the active site (Table S1 and Fig. S2).

For the rest of the work, we refer to the model derived from this sample as $SgrAI_{SP/F}^{CA}$ to indicate that this particular structure is filamentous and contains an intact SP, owing to the fact that the cleavage reaction was stalled through the use of Ca^2+^. We will contrast this model with a prior filamentous structure reported several years ago (16), which we refer to as $SgrAI_{no-SP/F}^{MG}$ to indicate that it contains Mg^2+^ but bound to DNA missing the SP at the cleavage site. We will also be referencing the previously reported structure (18) of a nonfilamentous dimeric form of SgrAI bound to Ca^2+^ and primary site DNA containing the SP, which will be referred to as $SgrAI_{SP/D}^{CA}$. Finally, a newly determined structure of nonfilamentous, DNA-free, and dimeric SgrAI bound to Ca^2+^ will be presented and discussed and referred to as $SgrAI_{apo/D}^{CA}$.

### Overview of the structure of activated filamentous SgrAI bound to DNA $\left(\mathrm{SgrA}I_{\mathrm{SP}/F}^{\mathrm{CA}}\right)$

**Figure 1:**
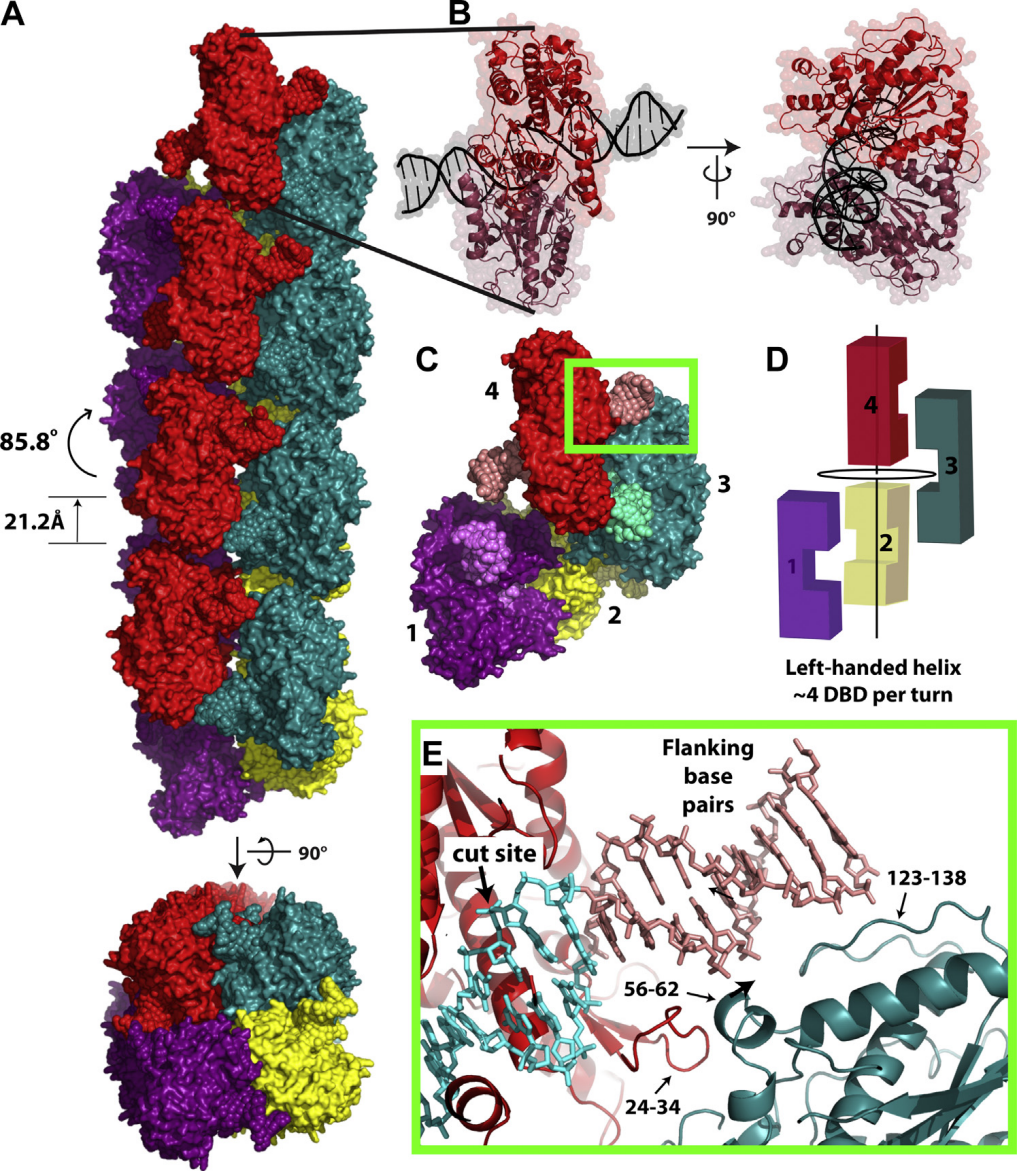
**Structure of DNA-bound SgrAI (DBD) and assembly into filaments.***A*, orthogonal views of $SgrAI_{SP/F}^{CA}$, with each DBD colored *magenta*, *yellow*, *teal*, or *red*. Protein chains are shown in *surface representation*, and DNA is shown in *spheres*. Each DBD is related to a prior DBD by a left-handed rotation of 85.8° and translation of 21.2 Å, leading to approximately four DBDs per turn. *B*, orthogonal views of a single DBD, with each protein chain of the SgrAI dimer in *light red* or *dark red* and bound DNA in *black*. *C*, four DBDs of the filament are shown to emphasize the left-handed helical twist between adjacent DBDs, with approximately four DBDs per turn. *D*, cartoon representation of four DBDs from the filament is shown in *C*. *Vertical line* represents the filament helical axis. DNA of each DBD is colored in slightly contrasting color. *E*, close-up view of the interactions between one chain of one DBD (*dark teal*) and that of a second DBD (*red* with DNA in *salmon*) from the *boxed region* in *D*. The 8 bp recognition sequence in the *red* DBD is colored in *cyan* with the cut site indicated.

**Figure 2:**
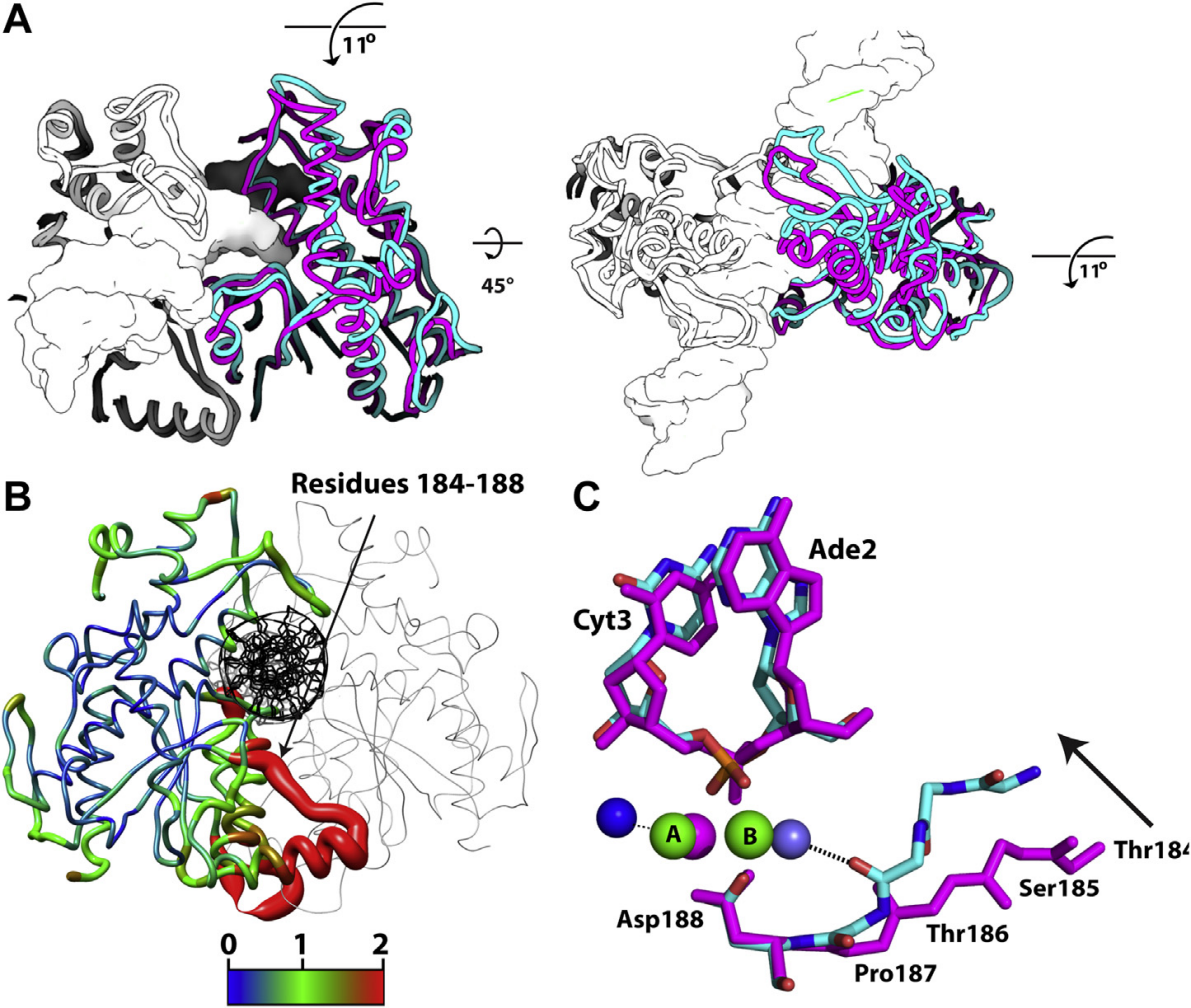
**Conformational changes upon filamentation lead to the creation of site B in the enzyme active site.***A*, superposition of $SgrAI_{SP/D}^{CA}$ (*white*/*pink*, Protein Data Bank code: 3DVO) and $SgrAI_{SP/F}^{CA}$ (*white*/*cyan*, this work) structures, both bound to primary site DNA. Side (*left*) and top (*right*) views are displayed. The *white* protomers are aligned, and the conformational change can be observed in the second protomer. It is evident that the second protomer chains are related by an ∼11° rotation about the long axis of the dimer. *B*, a single DBD from $SgrAI_{SP/F}^{CA}\;$ is displayed, and the left protomer in this DBD is colored by the RMSD to the corresponding protomer in the nonfilamentous form $SgrAI_{SP/D}^{CA}$. *Wider ribbon widths* indicate larger RMSD. Color legend is shown below (with RMSD in Å). The second chain, which was not used in the superposition, is colored *gray*. Bound DNA is colored in *black*. *C*, superposition of the active site within a single protomer of $SgrAI_{SP/F}^{CA}$ (*cyan*) or $SgrAI_{SP/D}^{CA}\;$ (*magenta*) using alpha carbons of three active-site residues (Asp188, Lys242, and Glu301). The shift in the segment containing Thr186 is apparent (*arrow*), which allows for hydrogen bonding to occur between the carbonyl oxygen of Thr186 and a water molecule ligated to the first shell of a Ca^2+^ ion bound in site B. DBD, DNA-bound SgrAI dimer.

The overall architecture of $SgrAI_{SP/F}^{CA}$ is shown in Figure 1*A*, which can be thought of as a left-handed helix with approximately four DBDs per turn (adjacent DBDs are rotated as shown by 85.8° and translated 21.2 Å along the helical axis). Two views of a single DBD are shown in Figure 1*B*, and the four DBD repeats are shown in Figure 1, *C* and *D*. The filament can be described as an ROO (14), since in principle DBDs can add to either end of the filament indefinitely. Lengths observed *via* cryo-EM, however, vary from two to more than 20, but this largely depends on the conditions used for sample and grid preparation (14). The structure of $SgrAI_{SP/F}^{CA}$ reinforces prior reports demonstrating the nature of the contacts between DBDs stabilizing the filament, which include protein–protein contacts between neighboring DBDs, as well as interactions between the SgrAI subunit of one DBD and the flanking DNA base pairs outside the 8 bp recognition sequence of a neighboring DBD (Fig. 1, *C* and *E*). Compared with the previously determined structure $SgrAI_{no-SP/F}^{MG}$, determined with DNA missing the SP moiety bridging nucleotides Ade2 and Cyt3, only small shifts in the position of the nucleotide Ade2 are found (Fig. S3*A*). As previously reported (16), a shift of the two subunits in the SgrAI dimer relative to each other is seen when comparing the filamentous and nonfilamentous DBD (Fig. 2*A*). Residues at the dimeric interface shift to accommodate the corresponding shift in position of the subunits. Figure 2*B* maps the RMSD of residues when comparing a single subunit in the two forms, with wider and redder portions indicating larger differences in residue position. Residues Thr184–Asp188 (Fig. 2*B*) bridge this interface and lead directly into the active site, where divalent cations bridge the protein–DNA interface (Fig. 2*C*). These residues move closer to the DNA-binding site in the filamentous conformation as compared with the nonfilamentous conformation (*arrow*, Fig. 2*C*), which has direct consequences for the mechanistic model underlying DNA cleavage that will be discussed later.

A total of three Ca^2+^ ions are located in each active site of the SgrAI dimer in sites A, B, and D (Fig. S3). Sites A and D are typically occupied in low-activity nonfilamentous structures of SgrAI (*i.e.*, $SgrAI_{SP/D}^{CA}$) (18, 19). However, occupancy of site B has important implications for the DNA cleavage mechanism, as predicted by the two-metal ion mechanistic model based on structure–function studies of many DNA nucleases (20, 23, 26, 27), discussed further below.

### DNA conformation

The conformation of the DNA bound to SgrAI in $SgrAI_{SP/F}^{CA}$ is very similar to that of $SgrAI_{no-SP/F}^{MG}$ with only a small shift of Ade2, likely because of the presence of the SP (Fig. S3*A*). DNA structural parameters of the 8 bp recognition sequence in $SgrAI_{SP/F}^{CA}\;$ are shown in Fig. S4 and compared with those of $SgrAI_{SP/D}^{CA}$ ($SgrAI_{SP/F}^{CA}$ is shown in *cyan*, $SgrAI_{SP/D}^{CA}\;$ is shown in *magenta*). Large differences are found between bps at the central step (between Cyt4 and Gua5, Fig. S4*C*) as well as in the tilt and twist at the outer 2 bps (Fig. S4, *D* and *F*). The large rise at the central base pair step was noted previously as likely originating from the accommodation of the 10° rotation of one SgrAI chain relative to another by the DNA (16). Structural differences at the outer 2 bps are of interest for understanding the special secondary structure activity of SgrAI; however, base stacking areas are more easily relatable to the energies of sequence-specific DNA structure because base stacking drives duplex stability (28, 29) and is sequence dependent (28, 30). Table S3 shows the base stacking areas of neighboring nucleotides in $SgrAI_{SP/F}^{CA}$ and $SgrAI_{SP/D}^{CA}$. As noted previously (16), the largest difference in stacking surface area of the DNA bases in the two structures is found at the second base step, between Ade2 and Cyt3 (*i.e.*, CACCGGTG), which shows a >250% increase in stacking surface area of the ring atoms in the filamentous form ($SgrAI_{SP/F}^{CA}$). The significance of this change will be discussed further in the context of secondary site activity.

### Protein–DNA interactions

The structures of $SgrAI_{SP/F}^{CA}$ and $SgrAI_{SP/D}^{CA}$ were compared for differences in interactions between SgrAI and the bound DNA. Fig. S5 shows overlays of the sequence-specific interactions between SgrAI and chemically distinct portions of the DNA bases in both conformations. Fig. S6 shows a map of the interactions with hydrogen bonds shown by *red* (for sequence-specific) or *dark gray* (for nonsequence-specific) lines. van der Waals interactions and close contacts that contribute to buried surface area are shown as *light gray lines*. No differences in sequence-specific protein–DNA interactions are observed; however, we note several other smaller differences (*green boxes*, Fig. S6). These include increases in buried surface area because of the close approach of Gln36 to Gua8 (6 Å^2^) in $SgrAI_{SP/D}^{CA}$, and in $SgrAI_{SP/F}^{CA}$, increases in buried surface area from the close approach of Ser244 to Gua5 (1.9 Å^2^) and from Gly284 to Cyt1' (10.5 Å^2^), as well as a new van der Waals contact between Gly284 to a flanking base (the Ade 5′ of Cyt1) (Fig. S6). Table S4 summarizes the total number of hydrogen bonds and van der Waals interactions, as well as buried surface area, at the protein–DNA interfaces of $SgrAI_{SP/F}^{CA}$ and $SgrAI_{SP/D}^{CA}$ (numbers given are for each half-site of the complex). Taken together, the major observation of the collective differences suggest an increase in buried surface area in the filamentous form by ∼200 Å^2^, increasing from ∼1400 Å^2^ for $SgrAI_{SP/D}^{CA}$ to ∼1600 Å^2^ for $SgrAI_{SP/F}^{CA}$ (Table S4). Both structures similarly show ∼30 hydrogen bonds to the DNA per protomer, but there was no change in the direct-readout hydrogen bonds to the DNA bases, with both structures maintaining 11 protein–base interactions per half-site. There appear to be slightly more van der Waals contacts in $SgrAI_{SP/D}^{CA}$ (∼160 per half-site) than $SgrAI_{SP/F}^{CA}$ (∼150 per half-site). These differences in contacts, along with other changes in the structures of SgrAI and DNA, are expected to contribute to the relative stabilities of the two DBD conformations. Because changes in structure at the outer 2 bps are of interest in understanding the secondary site activity of SgrAI, interactions involving those bps in each structural form are provided separately (Table S4). Only small changes are observed, with none involving sequence-specific contacts to the base atoms. Finally, the two filamentous structures, $SgrAI_{SP/F}^{CA}$ and $SgrAI_{no-SP/F}^{MG}$, were also compared using the aforementioned analyses, and no differences in protein–DNA contacts (with the exception of those mediated by the site B metal ion) were found consistent with the absence of any large-scale disruptions to the structure by the presence or the absence of the SP.

### The structure of DNA-free SgrAI $\left(\mathrm{SgrA}I_{\mathrm{apo}/D}^{\mathrm{CA}}\right)$ reveals novel conformational changes that shed light on secondary site specificity

**Figure 3:**
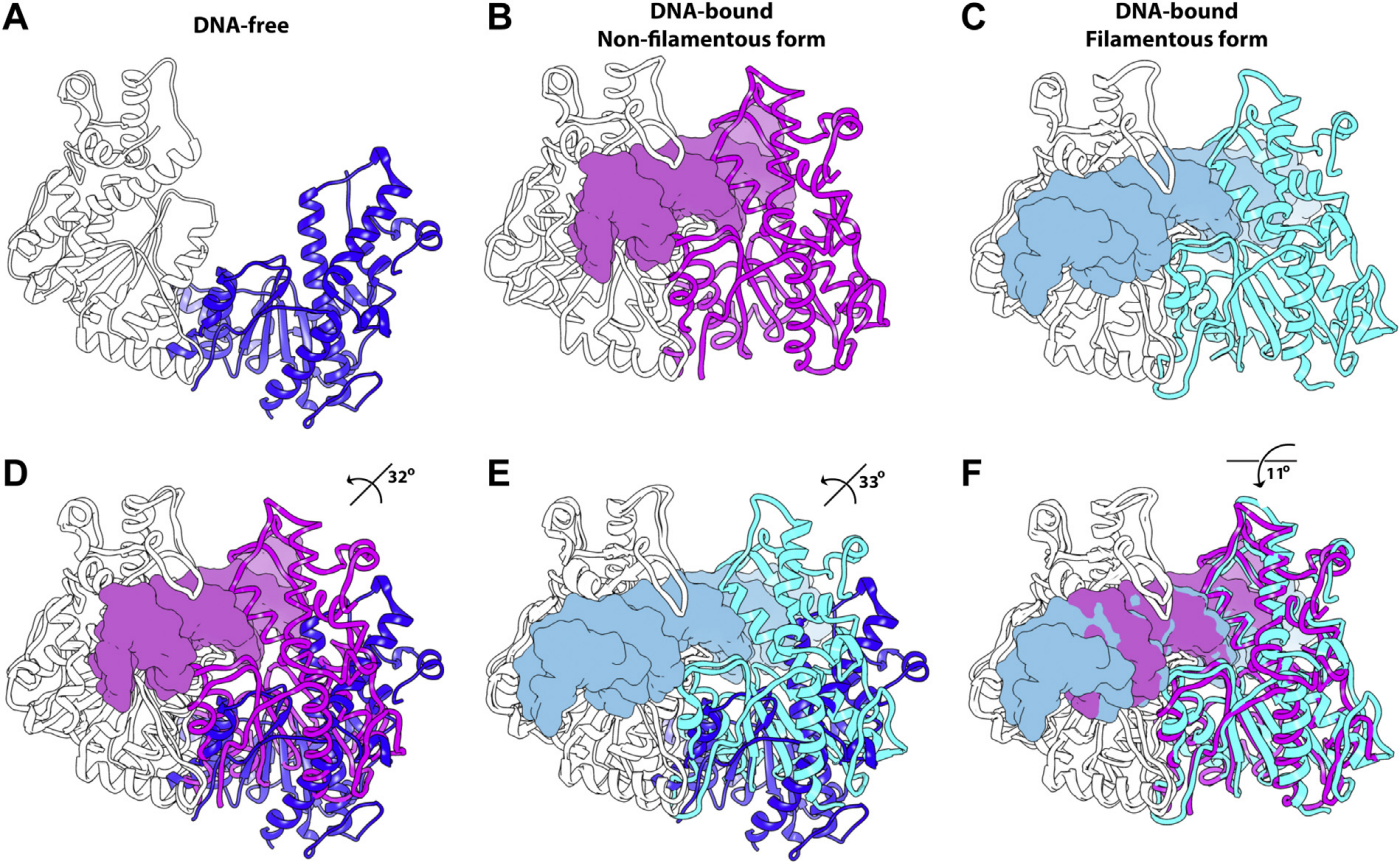
**Overall structure of**$\;\mathrm{SgrA}I_{\mathrm{apo}/D}^{\mathrm{CA}}\;$**and its comparison to DNA-bound forms of the enzyme.***A*, *ribbon diagram* of DNA-free dimeric SgrAI ($SgrAI_{apo/D}^{CA})\;$ with the two chains colored *white* or *blue*. *B*, ribbon diagram of dimeric DNA-bound SgrAI $\;(\;SgrAI_{SP/D}^{CA}$) with the two SgrAI chains colored *white* or *magenta*. DNA is shown as *light magenta* colored surface. *C*, ribbon diagram of filamentous DNA-bound SgrAI $\;(\;SgrAI_{SP/F}^{CA}$) with the two SgrAI chains is colored *white* or *cyan*, and bound DNA is shown in *light blue* surface rendering. *D*, single-chain superposition of the $SgrAI_{apo/D}^{CA}\;$ structure from (*A*) with that of $\;SgrAI_{SP/D}^{CA}\;$ from (*B*), emphasizing the change in relative position of the second chain. These chains are related by a rotation of 32° about an axis roughly perpendicular to the page. *E*, as in (*D*), but with $SgrAI_{apo/D}^{CA}\;$ and $\;SgrAI_{SP/F}^{CA}$ from (*A*) and (*C*), respectively. *F*, as in (*D*), but comparing $\;SgrAI_{SP/D}^{CA}\;$ and $\;SgrAI_{SP/F}^{CA}$ from (*B*) and (*C*), respectively.

In parallel efforts, we attempted to capture the filamentous form of SgrAI through crystallization experiments. To our surprise, we instead captured DNA-free SgrAI, without bound DNA. There are several reasons to which we can attribute this unexpected result. It may be because of nonspecific nuclease degradation of the DNA in the crystallization solution; alternatively, it may be due to the inherent difficulty in crystallizing heterogeneous species such as filaments of differing sizes. Regardless, these crystals diffracted to better than 2 Å, and we solved the structure by molecular replacement using a single chain from a prior crystal structure (Protein Data Bank [PDB] code: 3DVO) (18). Two copies of the chain were found per asymmetric unit arranged with twofold symmetry to produce a complete SgrAI dimer in its apo form, which we refer to as $SgrAI_{apo/D}^{CA}\;$ (Fig. 3*A*). The refinement to 2.025 Å resulted in a *R*_work_ and *R*_free_ of 20.2% and 24.6%, respectively.

The overall dimeric structure of $SgrAI_{apo/D}^{CA}$ is presented in Figure 3*A*. This structure shows a much more open configuration relative to DNA-bound forms, which are displayed in Figure 3, *B* and *C*. A superposition of $SgrAI_{apo/D}^{CA}\;$ and either of the two representative DNA-bound forms, including dimeric $SgrAI_{SP/D}^{CA}\;$ and filamentous $\;SgrAI_{SP/F}^{CA}$, indicate that the second chain is rotated by approximately 30° along an axis that is perpendicular to the dimeric twofold but roughly parallel with the helical axis of the bound DNA (Fig. 3, *D* and *E*). This opening of the dimer widens the nucleic acid–binding groove, which is expected to facilitate DNA binding by the enzyme. Importantly, the overall orientations of all forms of the enzyme: $SgrAI_{apo/D}^{CA}$, $SgrAI_{SP/D}^{CA}$, and $SgrAI_{SP/F}^{CA}$ differ; likewise, the axes of rotations between the three structures also differ. The two types of subunit rotations within the dimer relative to each other can be viewed as opening and closing of a clamshell between DNA-free and DNA-bound forms ($SgrAI_{apo/D}^{CA}$ *versus* either $SgrAI_{SP/D}^{CA}\;$ or $SgrAI_{SP/F}^{CA}$, Fig. 3, *D* and *E*) or more of a scissor-like movement between the two DNA-bound forms, one filamented and the other nonfilamented ($SgrAI_{SP/D}^{CA}$ *versus* $SgrAI_{SP/F}^{CA}$, Fig. 3*F*). To emphasize changes that occur within the subunit, Fig. S7, *A* and *B* show the RMSD displacement of C_α_ between $SgrAI_{apo/D}^{CA}\;$ and the two DNA-bound forms when only a single chain of the dimer is used in the comparison. The different position of the two subunits of the SgrAI dimer relative to each other in the different forms is accommodated by changes in positions of secondary structure elements and side chains at the dimeric interface. When both subunits are used in the superposition (with the current DNA-bound structure), the largest RMSD occurs at the outer edges of the structure (Fig. S4*C*). Collectively, these data highlight the configurational differences within the new DNA-free structure and shed light on the major conformational changes that must take place to accommodate the binding of nucleic acids.

The structure of $SgrAI_{apo/D}^{CA}\;$ also contains a divalent metal ion. A single Ca^2+^ is found bound to each subunit near the active site, in the site designated as site D (Fig. S8, see also Fig. S3*B* for the orientation of site D relative to sites A and B in $SgrAI_{SP/F}^{CA}$). This site is often found occupied by divalent cations in structures of SgrAI bound to DNA (18, 19). The Ca^2+^ bound to DNA-free SgrAI, $SgrAI_{apo/D}^{CA},$ is coordinated by six oxygen atoms arranged in an octahedral geometry, which are contributed by the side chains of Glu103 and Asn149, the main-chain carbonyl oxygens of Leu150 and Asp188, and two water molecules, respectively (Fig. S8). The role of this divalent cation-binding site is not clear, although its occupancy in the DNA-free structures suggests it may serve as a reservoir for Mg^2+^ prior to DNA binding, because Mg^2+^ in sites A and B bridge the protein–DNA interface. The site D divalent cation may also serve as a reservoir for binding to site B upon the change in conformation from low-activity to high-activity states in the DNA-bound form, when the site B pocket becomes favorable for metal ion binding. Similar proposals of moving metal ions in enzyme mechanisms have been made of other nucleases, such as EcoRV (31), DNA pol I (32), and the shift of the single Mg^2+^ in the DNA cleavage mechanism of APE1 (33).

**Figure 4:**
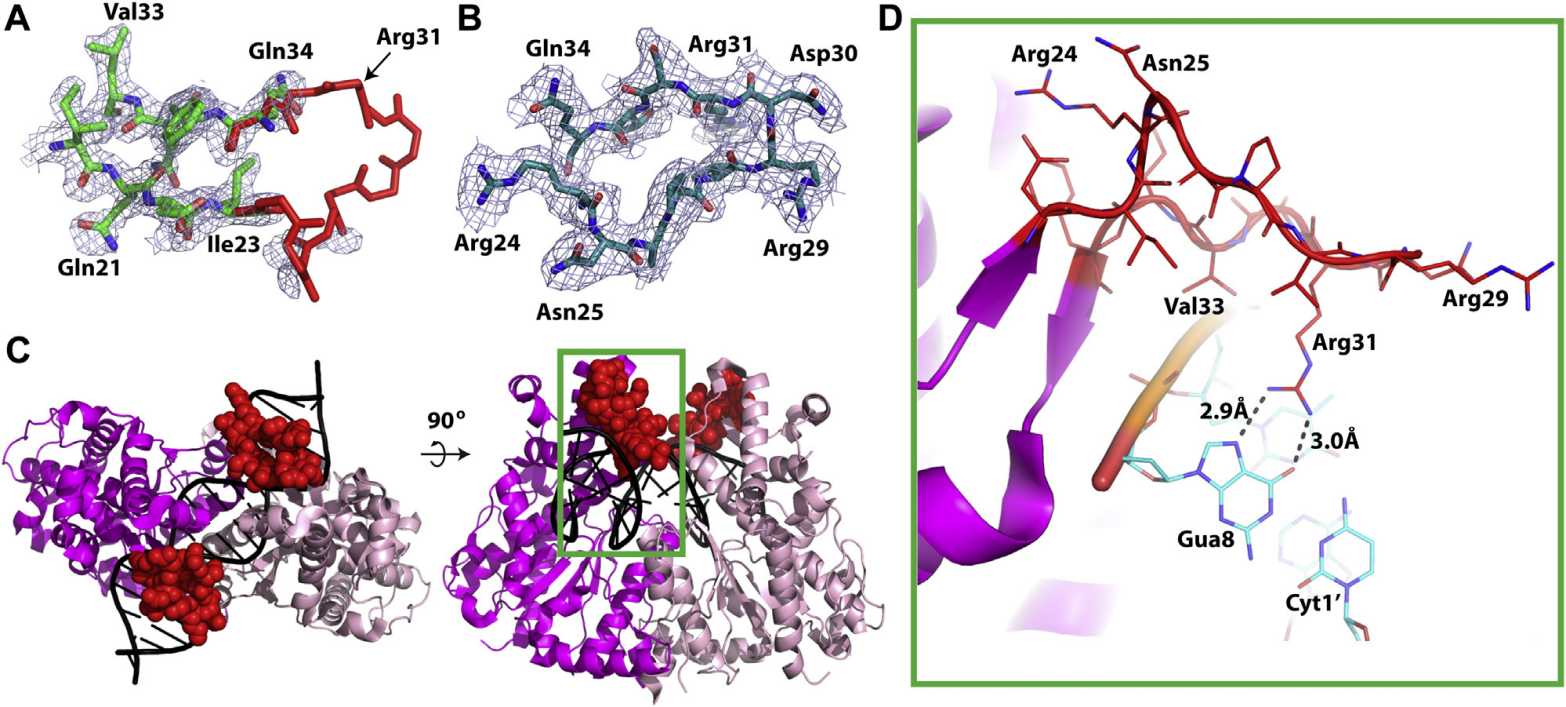
**Structural details of DNA-free nonfilamentous SgrAI**$\;(\mathrm{SgrA}I_{\mathrm{apo}/D}^{\mathrm{CA}}$). *A*, close-up view of the Arg31 loop and nearby atoms of $SgrAI_{apo/D}^{CA}\;$ (*green*, *blue*, and *red*) with residues Asp22–Gln34 from $SgrAI_{SP/D}^{CA}\;$ (*red*, Protein Data Bank: 3DVO) shown for reference, and 2*F*_o_–*F*_c_ map of $SgrAI_{apo/D}^{CA}\;$ contoured at 1σ showing little density for residues 24 to 33. Atoms in *red* are disordered in the DNA-free structure, but their location, as found in DNA-bound forms, is shown for reference. *B*, Arg31 loop of filamentous SgrAI $\left(SgrAI_{SP/F}^{CA}\right)$ and with map at 1σ showing well a defined map over this region. *C*, two views of a ribbon diagram of $\;SgrAI_{SP/D}^{CA}$ showing the position of the Arg31 loop of each subunit as *red spheres*, and each chain of the dimer in either *magenta* or *light pink*. *Green box* corresponds to view shown in (*D*). *D*, zoom in and cutaway of the *green boxed* area of (*D*) showing interactions between the Arg31 loop of the *magenta* subunit and the Gua8 base.

The structure of $SgrAI_{apo/D}^{CA}\;$ also reveals a region of the enzyme that is stabilized upon DNA binding. Residues Asp22–Gln34 within a loop region are disordered in the absence of DNA (Fig. 4*A*). In contrast, this loop is well ordered in crystal structures of SgrAI bound to primary site DNA (18) as well as cryo-EM structures of the filamentous DNA-bound form, including $SgrAI_{SP/F}^{CA}$ described here (Fig. 4*B*) (16). Figure 4*C* shows the position of this loop (*red spheres*) within the nonfilamentous SgrAI–DNA structure $SgrAI_{SP/D}^{CA}\;$ (PDB code: 3DVO) (18). This particular loop contains the residue Arg31, which makes two important hydrogen bonds to the guanine base of the last nucleotide of the SgrAI recognition sequence (CRCCGGYG) (Figs. 4*D* and S2*B*). We accordingly refer to this region as the Arg31 loop. It is evident that the Arg31 loop engages with the bound DNA, both in the major groove and along the sugar–phosphate backbone. This indicates that DNA binding induces loop ordering, which is necessary for Arg31 to interact with the guanine of the first base pair of the recognition sequence. Together with the 2.7 Å cryo-EM reconstruction of filamentous $SgrAI_{SP/F}^{CA}$, this second structure provides an important structural snapshot that sheds light on numerous aspects of SgrAI enzyme activity and sequence specificity. The novel insights will be discussed in the ensuing section.

## Discussion

### Implications of the substitution of Ca^2+^ for Mg^2+^ in the active site of SgrAI

**Figure 5:**
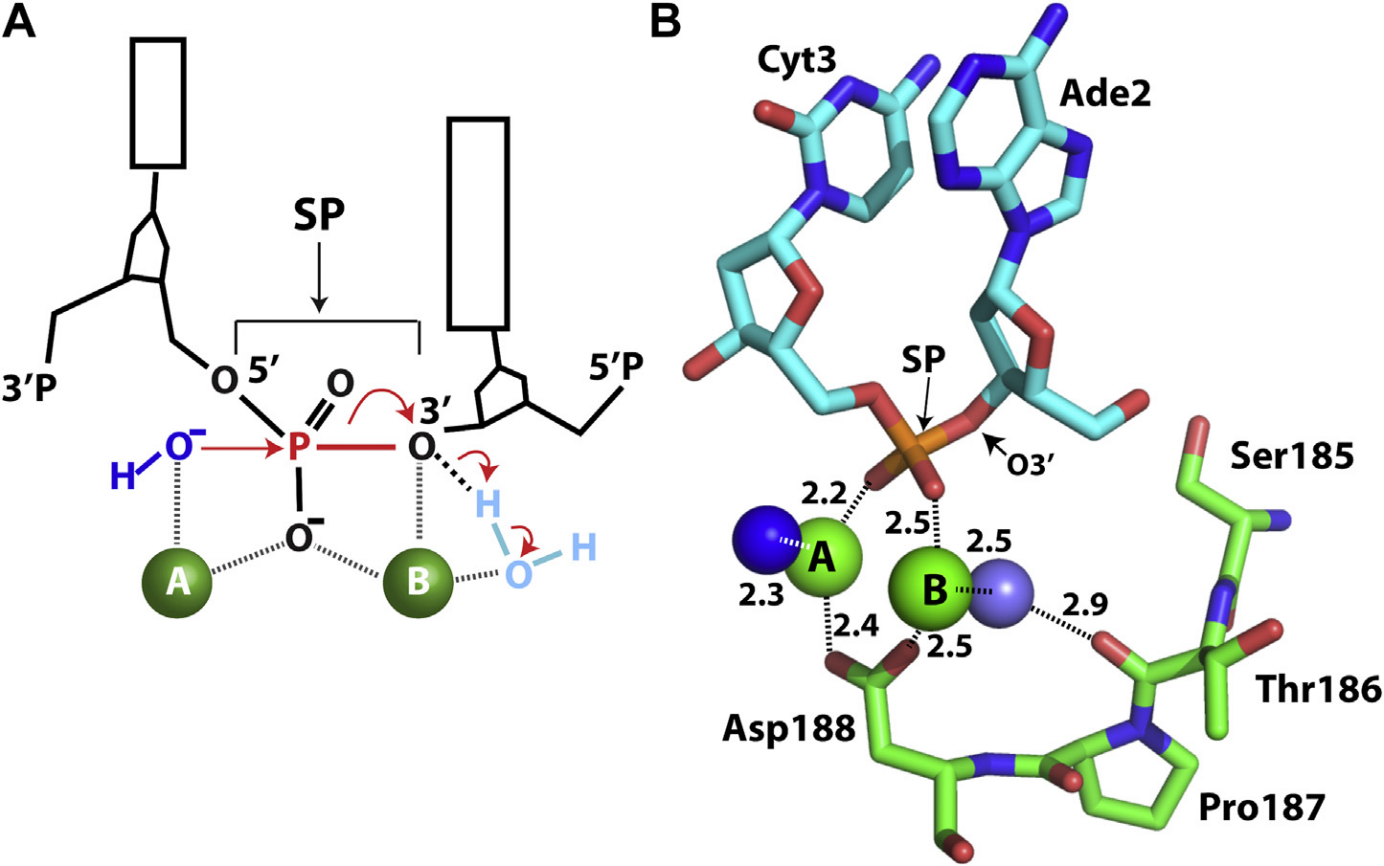
**Active-site configuration of**$\mathrm{SgrA}I_{\mathrm{SP}/F}^{\mathrm{CA}}\;$**in the context of the two metal ion mechanisms of catalytic DNA cleavage.***A*, schematic of the two-metal ion mechanism of catalytic DNA cleavage. Two divalent cations occupy sites A and B and ligate oxygen atoms of the nucleophile (shown as hydroxide in *dark blue*), one nonesterified oxygen of the scissile phosphate (SP), and in some enzymes also the 3′O leaving group as shown. Attack by the nucleophile on the phosphorus of the SP (*red*) is shown by a *straight red arrow*, and the bond to be broken between the phosphorus atom and the O3′ leaving group is shown as a *thick red line*. Electron shifts are shown as *curved arrows*. A water (*light blue*) ligated to the site B metal ion may donate a proton to stabilize the O3′ leaving group, which acquires a negative charge upon bond breakage. *B*, active site of chain B of $SgrAI_{SP/F}^{CA}\;$ showing Ca^2+^ ions in sites A and B. SP indicates scissile phosphate. Distances are given in Ångstrom. Relevant solvent molecules (putative nucleophile in *dark blue* and water molecule bridging to carbonyl oxygen of Thr186 in *light blue*) and nearby residues are indicated.

Reactions catalyzed by divalent cation-dependent DNA nucleases, as well as many other phosphoryl-transfer enzymes, are generally thought to utilize the two metal ion mechanisms originally proposed for alkaline phosphatase and DNA pol I (20, 21, 26, 34). In this model, the two metal ions (*i.e.*, divalent cations) are held in the enzyme active site on either side of the SP (Fig. 5*A*), with the A site metal ion serving to activate the nucleophile by lowering its p*K*_a_ and inducing deprotonation (22). In the case of divalent cation-dependent DNA nucleases, such as SgrAI, the nucleophile is a water molecule. The role of metal B is to stabilize the pentacovalent phosphorus transition state created upon nucleophilic attack and also to facilitate bond breaking by stabilization of the 3′O leaving group (through direct ligation or protonation by a metal ligated water) (22, 23, 26, 27, 34). In many enzymes, metal A also ligates the SP and thus also stabilizes the transition state, which bears an additional negative charge following nucleophilic attack (23, 27). The reaction is of the S_N_2 type, with inversion of configuration of the phosphate (35, 36, 37). Much of what we know about the two metal ion mechanism is derived from studies using X-ray crystallography; however, in order to capture an enzyme in a state prior to DNA cleavage, it is necessary to stall the reaction, which is typically accomplished using active site mutations, modifications to the DNA backbone, or metal ion substitutions. Since these substitutions prevent the enzymatic reaction from occurring, it is expected that some element(s) of the active site are either missing or structurally altered, whereas others should be preserved in their native conformation. Through the analysis of many such structures obtained at distinct stages of the enzymatic cycle or by using distinct tactics to stall the reaction, a comprehensive model for the active site mechanism can be constructed (25, 31, 38, 39, 40, 41, 42, 43, 44, 45, 46, 47, 48, 49). In the present work, we used a metal ion substitution (Ca^2+^ for Mg^2+^) to trap the active site prior to DNA cleavage. In our previous structure, we employed a DNA backbone substitution to capture the active site in the presence of Mg^2+^ (16). Taken together, the two distinctly stalled structures lead to a better understanding of the activated DNA cleavage mechanism employed by SgrAI. The absence of the SP in the prior structure (*i.e.*, $SgrAI_{no-SP/F}^{MG}$) captured Mg^2+^ binding at one of the two metal ion sites (site A), but no metal ion binding was seen in site B. In the current structure, utilizing Ca^2+^, we observe occupation of both expected metal ion–binding sites (Fig. 5*B*). Although both metal sites are occupied, and no other substitutions or mutations are present, the reaction is still stalled and the DNA remains uncleaved. Similar observations have been obtained in many studies of other DNA nucleases and phosphoryl-transfer enzymes (25, 26, 31, 42, 48, 50, 51, 52, 53, 54). The question of how Ca^2+^, despite its occupation of these two sites, inhibits the DNA cleavage (or other phosphoryl transfer) reaction has been the subject of many studies (26). Analysis of binding sites in small molecules and proteins indicates that both Ca^2+^ and Mg^2+^ prefer hard ligands such as oxygen but differ in their ionic radii (1.0 Å *versus* 0.72 Å for Ca^2+^ and Mg^2+^, respectively) and in their observed coordination numbers and geometries (55, 56, 57, 58). Mg^2+^ strictly prefers six oxygen ligands in an octahedral geometry, whereas Ca^2+^ can not only adopt the same coordination as well but also is commonly found with seven to eight ligands (55, 56, 57, 58). Elegant studies with RNaseH may provide clues to the differences in enzymatic activity with Mg^2+^ and Ca^2+^ in enzymes utilizing the two metal ion mechanisms. These studies showed that the two active-site Mg^2+^ ions must begin at ∼4 Å apart in the ground state and move closer toward each other during catalysis (possibly to a distance of 3.1 Å apart), but Ca^2+^ is too large, and the closest approach possible between two Ca^2+^ is estimated as 3.9 Å, which accordingly inhibits catalysis. A distance of 4.2 Å is observed in $SgrAI_{SP/F}^{CA}$ between the two Ca^2+^ ions, which is typical (26, 59, 60, 61). Hence, from the large body of work on divalent cation-dependent nucleases and other phosphoryl-transfer enzymes, it is expected that most features of the active site of SgrAI are preserved in the structure containing Ca^2+^, with the exception that the observed metal ion positions may be slightly mispositioned (likely no more than by ∼1 Å). This strategy for stalling the reaction enabled capturing multiple important features of activated SgrAI, discussed later.

### Identification of the site B metal ion and implications for the catalytic mechanism

Because SgrAI is dimeric, and since each protomer cleaves one strand of duplex DNA at a defined location, each protein subunit possesses an independent active site capable of binding the catalytic metal ion cofactors (18). Structures of the low-activity and nonfilamentous forms of SgrAI bound to primary site DNA (18) have two metal ions bound per active site, in site A as well as a distal site D but with no metal ion binding in site B. Since the two-metal ion mechanisms predict that both sites A and B are necessary for maximum cleavage activity, we proposed that the low enzymatic activity of the nonfilamentous form ($SgrAI_{SP/D}^{CA}$) of SgrAI was a direct result of the absence of site B occupancy (18). We also postulated that activation of the DNA cleavage activity results from filament-stabilizing conformational changes in SgrAI, which in turn stabilize Mg^2+^ binding in site B.

Our two earlier structures of filamentous SgrAI made clear that a conformational change does indeed occur within the enzyme in the context of the filament, leading to a distinct configuration of filamentous DBDs compared with nonfilamentous DBDs (14, 16). Superposition of one chain from each dimeric structure shows that the second chain is in a different position, characterized by a rotation of approximately 11° between the two. The rotational axis is perpendicular to the DNA helical axis as well as the dimeric two-fold axis, running along the long length of the dimer, such that the rotation can be thought of as a scissor-like shift between the two subunits (Fig. 3*F*). This conformational change appears to be stabilized by contacts made between DBDs within the filament. The earlier 3.5 Å structure of filamentous SgrAI provided additional details into the rearrangements of side chains and protein segments of each subunit at the dimeric interface that are necessary to accommodate the subunit–subunit rotation, as shown in Figure 2, *B* and *C* (16). These segments mostly occur at the dimeric interface. One of the segments that is characterized by a large displacement at the protein–DNA interface contains residues Gly181–Asp188, which emanate directly from the dimeric interface and lead to the enzyme active site within each subunit. Because the shifts in residues 181 to 188 occur very near the putative metal ion site B, we hypothesized that this shifted segment creates a pocket to stabilize the site B Mg^2+^ ion, specifically through the carbonyl oxygen of Thr186 (16). Such occupation of site B by Mg^2+^ would explain the faster DNA cleavage activity of SgrAI in the filamentous form (12). However, site B was not occupied in the earlier 3.5 Å structure of filamentous SgrAI bound to primary site DNA (*i.e.*, $SgrAI_{no-SP/F}^{MG}$) (16). We previously hypothesized that the absence of the phosphodiester bond linking the two nucleotides at the cleavage site (*a.k.a.* the SP) in the earlier structure resulted in the destabilization of Mg^2+^ binding in site B, despite the shift at Thr186 (27), since at least one oxygen ligand is expected to be provided by this phosphate (Fig. 5*A*). Consistent with our hypothesis, the current structure containing the SP indeed now shows a metal ion bound in site B (Figs. 2*C*, 5*B*, and S3, *A* and *B*). This Ca^2+^ ion forms interactions with the SP as expected, as well as with the carbonyl oxygen of Thr186, as predicted; however, the latter interaction is mediated through a water molecule rather than through direct ligation. In addition, a water molecule ligated to the site A Ca^2+^ is in the expected position of the nucleophile of the hydrolysis reaction catalyzed by SgrAI (*dark blue sphere*, Fig. 5*B*). Collectively, enzyme stabilization through the use of Ca^2+^ revealed novel interactions with the SP and provided one additional snapshot of the enzymatic reaction that enables us to piecemeal together a better picture of the cleavage mechanism.

### Structural changes proximal to secondary site substitutions within filamentous SgrAI provide insights into sequence specificity

**Figure 6:**
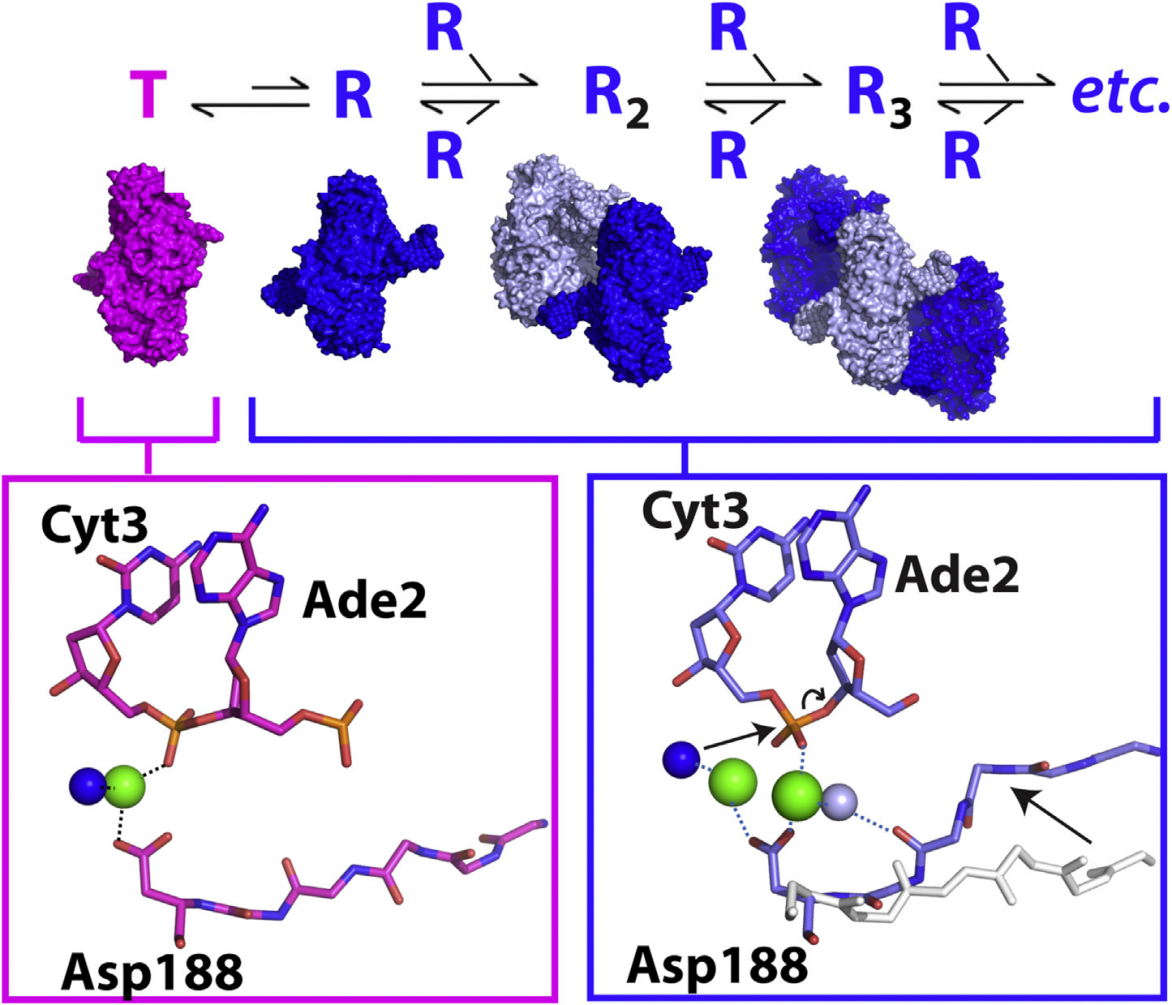
**Proposed mechanistic model of activation *via* filamentation of SgrAI by primary site DNA.***Top*, conformational equilibrium between the low-activity T state (*magenta*) and the high-activity R state (*blue*). Only the R state assembles into filaments. *Middle*, surface rendering of a single DBD of the T state (*magenta*, from $SgrAI_{SP/D}^{CA}$ of Protein Data Bank: 3DVO), a single DBD of the R state (*dark blue*, from $SgrAI_{SP/F}^{CA}$), and filamentous assemblies of two and three R-state DBDs (colored in different shades of *blue*). *Lower panels*, view of active-site arrangements in the representative T-state (*left*) and R-state (*right*) structures. A second divalent cation (*green sphere*) binds the active site in the R state in a site stabilized *via* a water molecule (*light blue sphere*) hydrogen bonded to the shifted protein main chain (shift indicated by *arrow* from *white* coordinates indicating T-state position to *blue* and *red* coordinates of the R state). Binding of the second divalent cation facilitates DNA hydrolysis. *Leftmost arrow* in *blue box* indicates nucleophilic attack of a divalent cation–bound water molecule (*dark blue sphere*). *Curved arrow* indicates bond breakage. DBD, DNA-bound SgrAI dimer.

Upon filamentation, SgrAI exhibits an apparent expansion in DNA cleavage sequence specificity. In the nonfilamentous low-activity form, SgrAI cleaves only its primary site sequences (CR|CCGGYG, R = A and G; and Y = C and T, and | indicates site of cleavage) but not its secondary site sequences (CC|CCGGYG, DR|CCGGYG, D = T, A, and G), despite the fact that both types of sites are bound by the enzyme with high affinity (12, 17). Filamentation of DNA-bound SgrAI stabilizes a change in enzyme conformation, which activates DNA cleavage activity on both primary and secondary sites (12, 16). However, SgrAI bound to secondary site DNA will not filament without the presence of SgrAI bound to primary site DNA (12). To explain this phenomenon, we created the model shown in Figure 6. In this model, SgrAI is in an allosteric equilibrium between two conformational states, a low-activity T state and a high-activity R state. The T state is intrinsically more stable, but the R-state conformation is favored within the filament. Because of mass action effects, increasing concentrations of SgrAI bound to primary site DNA also increase the concentration of R states, shifting the equilibrium to the right, that is, to favor filamentation, which in turn, increases the total concentration of R-state (activated) species. Crystal structures of nonfilamentous (*i.e.*, dimeric $SgrAI_{SP/D}^{CA}$) SgrAI bound to DNA exhibit the low-activity T state (18, 19). The important B-site metal ion (described previously) is absent in the T state, which provides an explanation for the low DNA cleavage activity in this conformation (18, 47) (*pink box*, Fig. 6). The R-state conformation is exhibited in structures of filamentous SgrAI, and as we now show, contains a metal ion in the B site, which is likely stabilized by conformational changes within each DBD that are, in turn, themselves stabilized by favorable interactions between DBDs within the filament (*blue box*, Fig. 6). Hence, under conditions favoring filamentation, DNA is cleaved more rapidly because of the higher total concentrations of R-state species. The T-state and R-state models of Figure 6 can also be used to understand the expansion of DNA cleavage sequence specificity (*i.e.*, the secondary site cleavage activity of SgrAI). If the T state is more favored when secondary site sequences are bound to SgrAI than when primary site sequences are bound, then SgrAI bound to secondary site DNA will be less inclined to filament, and DNA cleavage of secondary sites will be low, which is what is observed experimentally (12, 17). Filamentation can be driven by increasing the concentration of R states, which occurs when SgrAI bound to primary sites are also present; this increase in R-state species will drive the equilibrium of SgrAI bound to secondary site sequences to the right also, thereby inducing secondary site cleavage by SgrAI, which is again what is observed experimentally (12, 17). Therefore, this simple model can explain the observed behavior of SgrAI with the two types of sites (primary and secondary) and predicts that bp substitutions of the secondary site sequence, which occur at either the first or second base pair, perturb the R–T equilibrium toward the T state by differentially stabilizing the R and T state structures as compared with SgrAI bound to primary site sequences. This perturbation could be in the form of providing greater stability to the T state and/or lower stability to the R state (compared with SgrAI bound to primary site DNA).

To identify the origin of the energetic differences in T and R states containing different types of DNA sequences (*i.e.*, primary or secondary), we analyzed all available structures of SgrAI bound to both types of sites. At present, only a single structure of SgrAI bound to a secondary site sequence has been determined, which is in the low-activity nonfilamentous T state and contains a substitution in the second base pair (CCCCGGTG) (19). This structure showed no obvious changes in protein–DNA interactions or protein or DNA conformation compared with the T-state structure with primary site DNA (19). Both the high-activity R-state and low-activity T-state conformations ($SgrAI_{SP/F}^{CA}$ and $SgrAI_{SP/D}^{CA}$, respectively) are only available for SgrAI bound to a primary site DNA, CACCGGTG (16, 18). Comparing these two structures, we find no large changes in sequence-specific protein–DNA interactions at the outer 2 bps of the recognition sequence (nor at any bp in the 8 bp recognition sequence), which could explain a differential stabilization of R and T states by different base pair sequences at these positions (Figs. S5 and S6). However, we do observe changes in the DNA structure itself (Figs. S3 and S4 and Table S3) as well as a close approach of a segment of SgrAI to the outer base pair of the 8 bp recognition sequence in only the R-state structure. Both these may play roles in the secondary site activity of SgrAI, as discussed further.

Previously, we noted the change in stacking between the second and third bases of the bound primary site DNA (CACCGGTG) when comparing R and T states (Table S3 and Fig. S9) (16). We argued that this change in stacking could lead to a change in R and T state energies that is also dependent on base identity, since stacking energies drive DNA duplex stability (28, 29) and are also heavily sequence dependent (28, 30). Such an indirect readout mechanism would explain the differences in R and T state energies between primary sites and secondary sites of the sequence CCCCGGYG, which are substituted in the second position. Experimentally determined values of base stacking indicate that an Ade–Cyt or Gua–Cyt base step (found in primary site sequences) provides greater stacking energy than a Cyt–Cyt step as found in this class of secondary site sequences (30). Hence, the gain in stacking area, and by extension stacking energy, at the second base step in the R state provides more stabilization to complexes bound to primary site sequences than to those bound to secondary site sequences. This gain differentially affects the R–T equilibrium in DBD with the two types of sequences; though both still favor the T state, the R state is disfavored more by DBD containing secondary site sequences than those containing primary site sequences, consistent with the lower propensity of DBDs with secondary site sequences to filament (Fig. 6). In the current structure of $SgrAI_{SP/F}^{CA}$ at 2.7 Å, the large change in stacking surface area at the second base step previously observed in the $SgrAI_{no-SP/F}^{MG}$ structure is preserved (Table S3). The observed difference in base stacking is significant and not due to coordinate error, as shown by the superior fit of the shifted base step to the map of the $SgrAI_{SP/F}^{CA}\;$ structure. Fig. S10 shows an overlay of the Ade2–Thy7′ bp (after superposition of base atoms of Cyt1–Gua8′ of both structures) and the map from $SgrAI_{SP/F}^{CA}$ (the R state). It is clear that the $SgrAI_{SP/F}^{CA}\;$ structure (the R state, with carbon atoms in *blue*) fits the map significantly better than the $SgrAI_{SP/D}^{CA}$ (the T state, with carbon atoms in *pink*) structure, as portions of the $SgrAI_{SP/D}^{CA}$ structure emerge from the map (*arrows*, Fig. S10). Furthermore, the correlation coefficient between the model and map is a recognized measure of model quality and map-model fit (62) and is significantly higher for the coordinates of this bp from the $SgrAI_{SP/F}^{CA}$ (0.86) structure than for that of the $SgrAI_{SP/D}^{CA}$ (0.75) structure, when comparing to the $SgrAI_{SP/F}^{CA}$ cryo-EM map. The shift in bp position between the two structures is also significant relative to the predicted coordinate error; the predicted coordinate error of a well-refined crystallographic structure at 2.7 Å is 0.2 to 0.25 Å (63), but the shift of the base centroids of this bp is observed to be 1.0 Å, considerably larger than the estimated coordinate error. Hence, our studies predict that the origin of the secondary site activity of SgrAI with sites substituted at the second bp of the 8 bp recognition sequence results from the distortions of bound DNA, specifically between the second and third nucleotides, which in turn disfavor the R state to a greater degree than SgrAI bound to primary site DNA.

**Figure 7:**
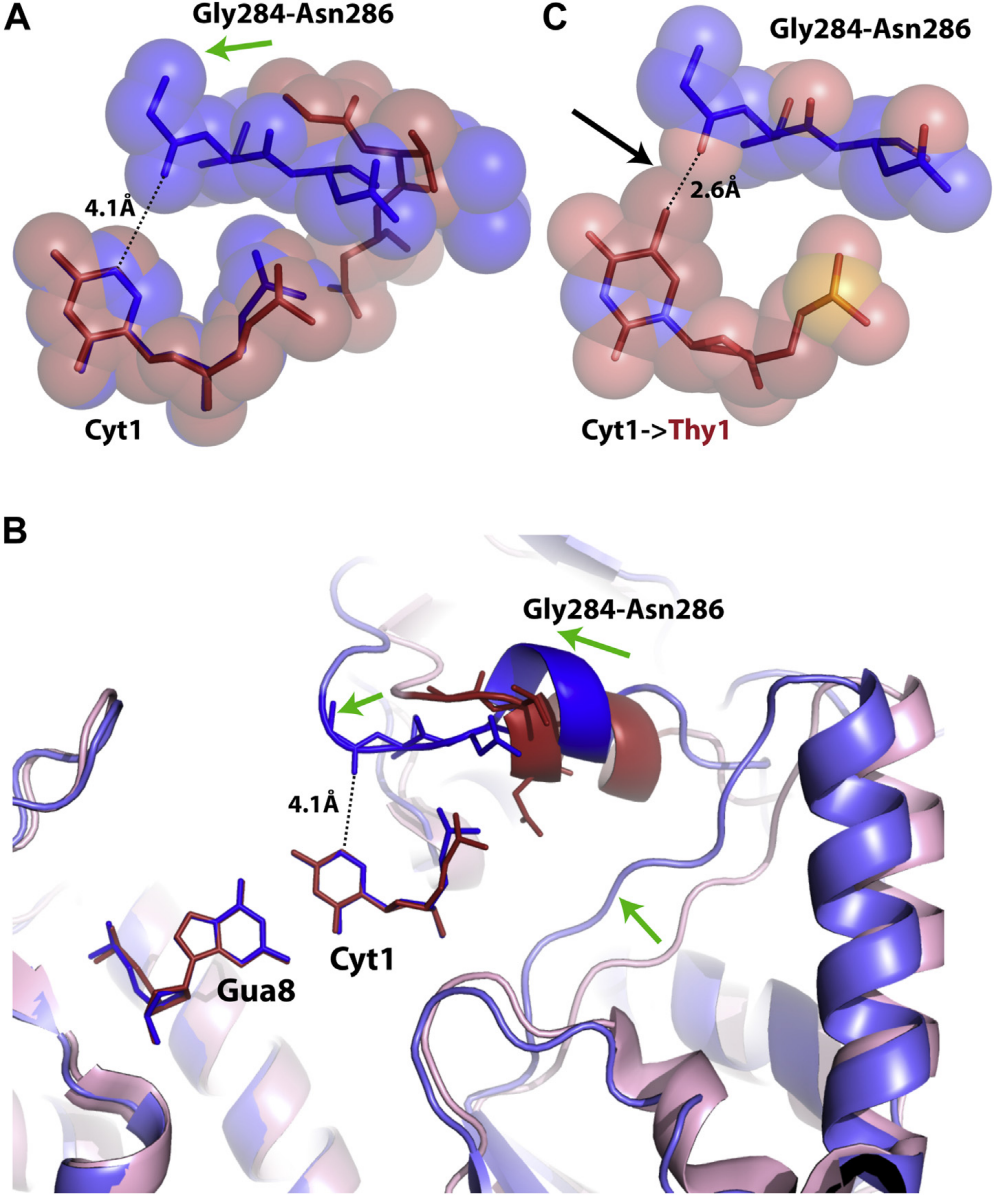
**Residues 284 to 286 are shifted closer to the outer base pair of the recognition sequence in the activated filamentous structure.***A*, superposition of the base atoms of Cyt1:Gua8 in one half-site of the DNA recognition sequence in $\;SgrAI_{SP/F}^{CA}$ (*blue*) and $\;SgrAI_{SP/D}^{CA}\;$ (Protein Data Bank code: 3DVO, *red*) showing the difference in the position of residues Gly284–Asn286 (*green arrow*). *B*, as in (*A*), but emphasizing changes in positioning of segments proximal to the DNA because of the rotation of one subunit of the dimer relative to the other (*green arrows*), which in turn brings residues Gly284–Asn286 closer to the first base pair of the recognition sequence. *C*, as in *A*, but Cyt is replaced with a Thy in the first base pair of the recognition sequence, revealing a potential steric overlap (2.6 Å) of its 5-methyl to the carbonyl oxygen of Gly284 (*black arrow*).

Regarding secondary site sequences substituted in the first bp, as in the DRCCGGYG (D = A, G, or T) class of secondary sequences, we propose a different mechanism of preferential T-state stabilization. Though some difference in base stacking at the first base step in R-state and T-state structures is observed, it is of a smaller amount than at the second base step (Table S3), and the large number of substitutions possible at the first bp (each with distinct associated stacking energies) makes a unified indirect readout mechanism unlikely. Also, substitution at the first bp weakens the binding affinity for SgrAI by as much as 15-fold (12, 64), since the hydrogen bonds to Gua8 (the base pairing partner of the first nucleotide of the recognition sequence, Figs. S2*B* and 4*D*) cannot form. But this is true of both R and T states, which both contain the Arg31–Gua8 interaction, and therefore loss of this interaction should not be expected to affect the equilibrium between them. However, we have found two structural changes that may explain the observed behavior of SgrAI with this class of secondary site sequences. First, we propose that residues Gly284–Asn286, near the first base pair of the recognition sequence, play an important role in discriminating between primary and secondary site DNAs. From the current work, it is clear that the position of residues Gly284–Asn286 is different in the representative T-state and R-state structures (Fig. 7, *A* and *B*). Specifically, the conformation observed in $SgrAI_{SP/F}^{CA}$, containing the SP, brings the carbonyl oxygen of Gly284 within 4.1 Å of the C5 atom of Cyt1 of the DNA (*blue*, Fig. 7*A*). This change in position is a direct consequence of the large shift in subunit–subunit orientation between the T and R states (16) (Figs. 7*B* and S11). If Cyt1 is substituted with a Thy, as in the secondary sequence TRCCGGYG, such a substitution would place the C5-methyl group of Thy within ∼2.6 Å to the carbonyl oxygen of Gly284, resulting in a steric overlap, as the expected distance between a methyl group and a carbonyl oxygen is ∼3.8 Å (Fig. 7*C*). Steric issues from substitution to Ade or Gua are less clear (Fig. S12), but the close approach (3.8 Å) of the N7 of the purine rings with the carbonyl oxygen of Gly284 may interfere with hydrogen bonding to water molecules, leaving the hydrogen-bonding groups unsatisfied and therefore providing less stability to the structure. Hence, this close approach of the segment containing Gly284 in the R state may contribute to disfavoring the R-state conformation over the T-state conformation when bound to secondary sites containing substitutions at the first base pair of the recognition sequence.

**Figure 8:**
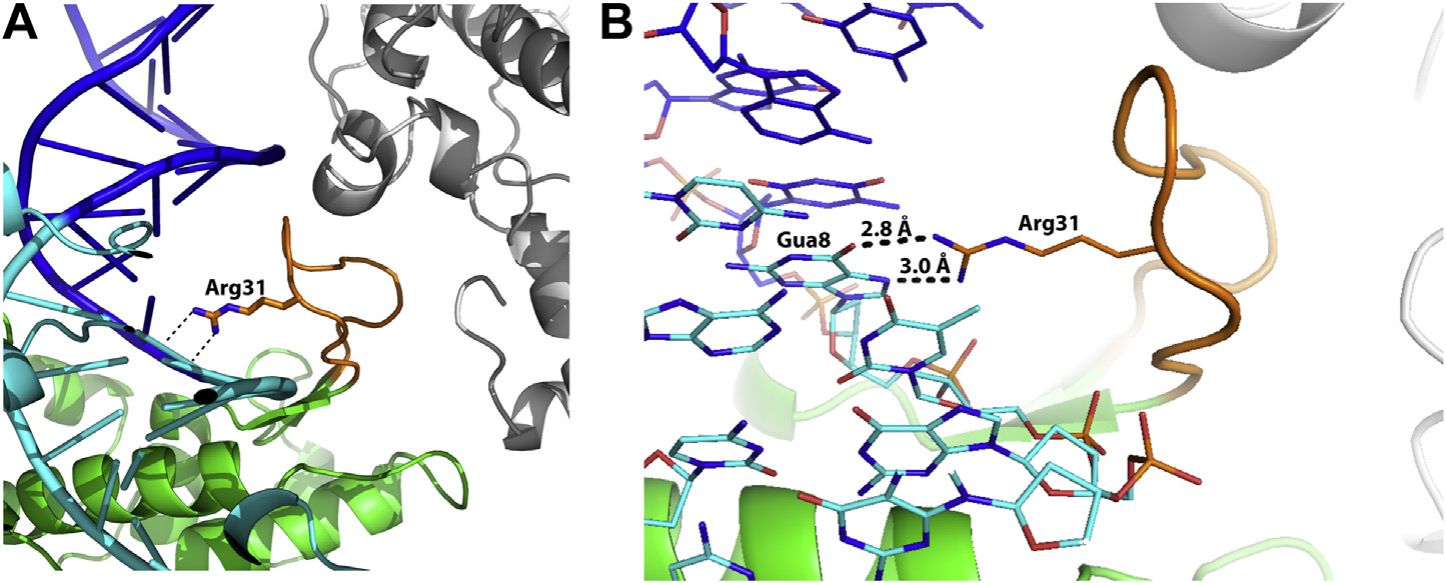
**The Arg31 loop in protein–protein contacts within the filament.***A*, residues Asp22–Gln34 (*orange*) contain Arg31, which not only makes critical recognition contacts to Gua8 but also forms protein–protein contacts to a neighboring copy of SgrAI (*white*) in the filament. Ordering of this loop may require recognition of the outer G of the recognition sequence (*i.e.*, Gua8, *dashed lines* show hydrogen bonds). When such interactions cannot be made to the outer base pair, because of sequence substitution in secondary sequences, this loop may be disordered, requiring a disorder-to-order entropic cost upon filament formation. In the figure, the DNA is colored *dark blue* (flanking base pairs) and *light blue* (core 8 bp recognition sequence). *B*, close-up of Arg31–Gua8 interactions showing hydrogen-bonding distances.

The DNA-free SgrAI in its dimeric apo form ($SgrAI_{apo/D}^{CA}$) may also shed light on the secondary site activity, particularly with substitutions at the first bp of the recognition sequence. In this structure, the residues Asp22–Gln34 within the Arg31 loop are disordered (Fig. 4*A*), but these same residues are well ordered in all structures of SgrAI bound to primary site DNA, both dimeric and filamentous forms (Fig. 4, *B*–*D*) (16, 18). Arg31, which makes two important hydrogen bonds to Gua8 (the last nucleotide of the SgrAI recognition sequence (CRCCGGYG) (Figs. 4*D* and S2*B*), is found within this loop. These results indicate that DNA binding induces an ordering of loop Asp22–Gln34, which includes the interaction between Arg31 and Gua8. The result is twofold: first, there is an enthalpic gain characterized by the newly formed hydrogen bonds, van der Waals interactions, and salt bridges between protein and DNA; at the same time, there is also an unfavorable entropic cost that is incurred because of the necessity to order these residues from a disordered and higher entropy state. For secondary sequences substituted in the first bp (*i.e.*, DRCCGGYG, D = A, G, or T), the base pairing partner of the first nucleotide (namely a T, C, or A) will not be capable of making the same hydrogen bonds to Arg31 as the primary sequence. As a result, the Arg31 loop may remain partially or even completely disordered, and as such, differentially affect the filamentation of DBDs containing secondary site sequences, since this loop forms a large interaction interface with a neighboring DBD in the filament (*orange segment*, Fig. 8). When the Arg31 loop is already ordered appropriately, for example, by engaging with primary site DNA, this part of the DBD is preformed and may associate with other DBDs in the filament with little entropic cost. However, when a DBD has a disordered Arg31 loop, the Arg31 loop must undergo the disorder-to-order transition to form the correct interface with other DBDs within the filament. Hence, in such cases, the entropic cost of ordering the Arg31 loop will occur at the filament assembly step, rather than the DNA-binding step, and as such disfavor filamentation.

### The mechanism of activation and expansion of substrate specificity in the SgrAI system

**Figure 9:**
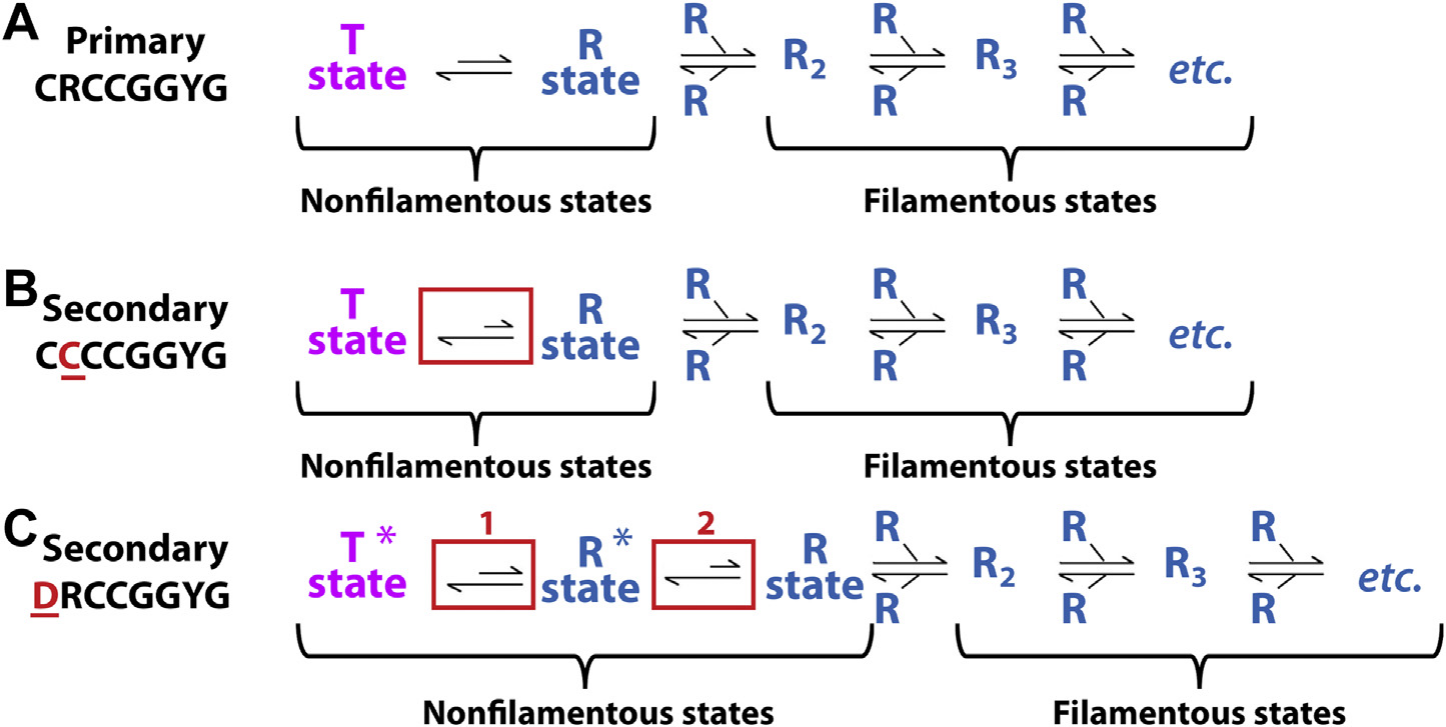
**Mechanistic model for primary and secondary site activity of SgrAI.***A*, equilibria between structural states when SgrAI is bound to primary site DNA. The T state is intrinsically favored, but filamentation shifts the equilibria right to favor the R state, which possesses higher DNA cleavage activity. *B*, as in (*A*), but when SgrAI is bound to secondary site sequences substituted in the second base pair (*red text and underlined*). The differences in DNA stacking energy at the substituted base change the relative stabilities of the T and R states to favor T to a greater extent (*red box*, compare to equivalent step in *A*). SgrAI bound to this secondary site will not form filaments in the absence of SgrAI bound to primary site DNA, which contributes R states to the equilibrium, driving it toward the right in the reaction scheme. *C*, as in *A*, but for SgrAI bound to secondary site sequences substituted in the first base pair (*red text and underlined*). The inability to form hydrogen bonds from Arg31 to this bp results in disorder of the Arg31 loop (indicated by the *asterisk* in T^∗^ and R^∗^ states). The transition to the R^∗^ state from the T^∗^ state is disfavored to a greater extent than when primary site is bound (*red box* 1, compare with equivalent step in (*A*) because of a close contact between SgrAI and the first base of the recognition sequence (*red box* 1). In addition, an unfavorable disorder-to-order transition is necessary to form the filament competent R state from the R^∗^ (*red box* 2). Hence, SgrAI will not filament and will not cleave DNA when bound to this sequence, except in the presence of SgrAI bound to primary sites, which contribute R states driving the equilibrium to the right in this scheme.

The structure of $SgrAI_{SP/F}^{CA}$ described herein bears out our prior prediction of occupation of the site B metal ion in the activated and filamentous form of SgrAI (16). The difference in positioning of Thr186 between this conformational state and structures of nonfilamentous SgrAI appears to play a role in stabilization of site B occupancy as predicted, although we see here that the interaction with the site B metal ion occurs through a bridging water molecule rather than *via* direct ligation (Figs. 2*C* and 5*B*). In agreement with prior work, we find changes in DNA structure that may underlie the unusual modulation of DNA sequence specificity of SgrAI upon filamentation (16). The mechanistic model shown in Figure 9 summarizes these conclusions. Filamentation is driven when R states are occupied significantly as occurs with SgrAI bound to primary site DNA (Fig. 9*A*). The T state is favored over the R state because of the balance of enthalpic and entropic contributions within the protein, the DNA, and between the protein and bound DNA, in each conformation. Bp substitutions in the DNA recognition sequence shift the R–T equilibria to the left, to favor the T state, resulting in a lowered propensity to filament and much decreased DNA cleavage activity. In the case of substitutions in the second base pair of the recognition sequence, we propose that the energetics of DNA structure are responsible for this shift (*red box*, Fig. 9*B*). For substitutions occurring in the first base pair, we hypothesize that an altered ground state (T^∗^) will be exhibited because of the disordered Arg31 loop near the site of the substitution (Fig. 9*C*, see also Fig. 4*A*). If the loop remains disordered in the R state (R^∗^), an additional step (*boxed step* 2, Fig. 9*C*) will be required to order the loop in order for filamentation to occur. We also propose that the close approach of Gly284 to the first bp also contributes to disfavoring the R states in this case (*boxed step* 1, Fig. 9*C*, see also Fig. 7*B*), and as a result of both these mechanisms, filamentation is diminished. Cleavage of secondary sites by SgrAI in the presence of primary sites is explained by the shifting of equilibria to the right by the increased concentrations of R states, which then drive filamentation of SgrAI bound to either type of secondary site (Fig. 9, *B* and *C*).

The aforementioned analyses are based on three structures, that of nonfilamentous SgrAI bound to secondary site DNA substituted in the second position, representing the T state of Figure 9*B*, and those of SgrAI bound to primary site DNA, both filamentous representing the R state (Fig. 9*A*) $(SgrAI_{SP/F}^{CA}$) and nonfilamentous representing the T state (Fig. 9*A*) ($SgrAI_{SP/D}^{CA}$). Our predictions regarding secondary site effects on equilibria assume similar conformations of SgrAI bound to the two types of sites in the R state as well as for the T state of SgrAI-bound secondary site substituted in the first base pair (Fig. 9*C*), with the exception of the predicted disorder in the Arg31 loop of the latter (resulting in T^∗^). Having experimental structures of SgrAI bound to both types of secondary site in the filamentous (R) state, as well as to the secondary sites substituted in the first base pair in the T state, will be necessary to test these predictions.

## Experimental procedures

### Protein preparation

SgrAI enzyme was prepared as previously described (17). Briefly, SgrAI was expressed in BL21 (DE3) *Escherichia coli* (which also contain a constitutive expression system for the methyltransferase MspI.M) overnight at 22 °C. Cells were lysed in lysis buffer (100 mM sodium phosphate buffer, pH 8, 800 mM NaCl, 1 mM 2-mercaptoethanol, and 1 mM PMSF) using an Avestin Emulsiflex C3 (Avestin, Inc) and centrifuged for 70 min at 11,000 rpm to remove cell debris. SgrAI was isolated by incubation with Talon resin (Clonetech, Inc) in lysis buffer for 45 min, followed by washing of the resin with wash buffer (100 mM sodium phosphate buffer, pH 8, 300 mM NaCl, and 1 mM 2-mercaptoethanol) and high salt buffer (100 mM sodium phosphate buffer [pH 8], 2 M NaCl, and 1 mM 2-mercaptoethanol), and finally eluted using elution buffer (100 mM sodium phosphate buffer, pH 8, 300 mM NaCl, 250 mM imidazole, and 1 mM 2-mercaptoethanol). Then purified protein was dialyzed into heparin buffer A (100 mM sodium phosphate buffer, pH 8, 50 mM NaCl, 0.1 mM EDTA, and 1 mM 2-mercaptoethanol) to purify with heparin FF chromatography (GE Healthcare Biosciences) and a gradient of heparin buffer B (100 mM sodium phosphate buffer, pH 8, 1 M NaCl, 0.1 mM EDTA, and 1 mM 2-mercaptoethanol). Further purification was performed using size-exclusion chromatography (Superdex-200; Cytiva, Inc) with size-exclusion chromatography buffer (25 mM Tris–HCl, pH 8, 150 mM NaCl, and 1 mM DTT). Purified SgrAI was concentrated and stored in single-use aliquots at −80 °C in buffer containing 50% glycerol. Enzyme purity was assessed using Coomassie blue staining of SDS-PAGE and assessed to at least 99% purity.

### DNA preparation

Oligonucleotides were prepared synthetically by a commercial source and purified using C18 reverse-phase HPLC. The concentration was measured spectrophotometrically, with an extinction coefficient calculated from standard values for the nucleotides (65). Equimolar quantities of complementary DNA were annealed by heating to 90 °C for 10 min at a concentration of 1 mM, followed by slow cooling to room temperature. The sequence of the DNA used in SgrAI–DNA preparations is shown below (*red* indicates the SgrAI primary recognition sequence):

40-1-top 5′-GATGCGTGGGTCTTCACACCGGTGGATG CGTGGGTCTTCA-3′

40-1-bot 3′-CTACGCACCCAGAAGTGTGGCCACCTAC GCACCCAGAAGT-5′

PC-4-top 5′-CGTGGGTCTTCACA-3′

PC-4-bot 3′-GCACCCAGAAGTGTGGCC-5′

### Sample preparation for EM $\left(\mathrm{SgrA}I_{\mathrm{SP}/F}^{\mathrm{CA}}\right)$

The purified SgrAI protein was subjected to a gel filtration with a Superdex 200 Increase 10/300 GL(GE) size-exclusion column just before grid preparation. The column was pre-equilibrated in buffer (25 mM Tris–HCl [pH 8.0], 150 mM NaCl, and 1 mM Tris(2-carboxyethyl)phosphine). Peak fractions were analyzed by SDS-PAGE. Pure fractions were pooled and concentrated using an Amicon 5 ml 10,000 molecular weight cutoff centrifugal concentrator (Millipore Sigma, Inc) to a concentration of 6 μM SgrAI dimer.

SgrAI–DNA filaments were assembled by mixing 10 μl of 6 μM SgrAI with 1 μl of 500 μM dsDNA in H_2_O, 1.2 μl of 100 mM CaCl_2_, and incubation at room temperature for 1 h. The final concentrations were 4.9 μM SgrAI, 41 μM dsDNA (dsDNA:SgrAI is 8.4:1), and 10 mM Ca^2+^. After incubation, the sample was centrifuged at 12,000 rpm for 1 min to remove large aggregates before applying to R1.2/1.3 gold UltrAufoil grids, Au 300 mesh (Quantifoil). Cryo-EM grids were prepared by manually freezing using a manual plunger in cold room at 4 °C. The grids were clipped and subsequently stored in liquid nitrogen for future data acquisition.

### Cryo-EM data collection

Cryo-EM movie frames were collected on a Titan Krios transmission electron microscope (Thermo Fisher Scientific) operating at 300 keV. A K2 summit direct detector (Gatan) with a Gatan Imaging Filter BioQuantum energy filter was used to record the movies using a slit width of 30 eV for data collection. Data collection was performed using the Leginon software (66, 67) at a magnification of 165,000×, corresponding to a pixel size of 0.83 Å/pixel in microprobe energy-filtered transmission electron microscopy mode. Movies composed of 75 frames were collected in counting mode on the K2 detector over 6 s exposure (80 ms per frame). The total fluence was 30.5 e^−^/Å^2^ at a rate of 3.5 e^−^/pix/s. All imaging parameters are summarized in Table S1.

### Cryo-EM image analysis

The movie frames were motion corrected and dose weighted using MotionCor2_1.4.0 (68) on six-by-six patch squares and using a *B*-factor of 100. The gain reference used for MotionCor2_1.4.0 was generated by using the Sum_all_tifs program, which is packaged into the cisTEM image processing suite (69). The motion corrected micrographs were imported into cryoSPARC, version 3.2.0 (Structura Biotechnology Inc) (70), which was then used to perform patch contrast transfer function (CTF) estimation and particle selection. Manually picked particles were initially extracted with a box size of 320 pixels and then used to perform 2D classification. The best selected class averages from this initial round of 2D classification were used as 2D templates for template-based particle selection in cryoSPARC. For template picking, we set a particle diameter of 100 Å with an overlap that did not allow any two picks to be closer than 0.2 units of particle diameter in distance or about the rise of a single subunit. About 278,865 particles were extracted with a box size of 320 pixels after inspection of particle picking. Reference-free 2D classification was used to identify filamentous particles, which was performed immediately after particle extraction. After several rounds of 2D classification, 220,067 particles remained. We selected class averages based on the appearance of filamentous particles with good features. The best 2D classes were selected for generating an *ab initio* reconstruction, using two classes as input. The *ab initio* with clearly defined features consistent with SgrAI was selected for subsequent downstream analysis. The clean particle stack from 2D was then subjected to homogeneous helical refinement in cryoSPARC, which resulted in a ∼3.2 Å map, according to the Fourier shell correlation (FSC) criterion obtained using a fixed threshold of 0.143. Following homogeneous helical refinement, we performed one round of per-particle CTF refinement and one round of global CTF refinement, which improved the resolution to ∼3.1 Å. At this point, the particles were imported into Relion 3.1 for particle polishing (71, 72). Particle polishing was performed in Relion using default parameters. Subsequently, to take advantage of the ability to account for multiple unique asymmetric units within the selected particles, we performed the final reconstruction in Relion, which provides this option. The final refinement was performed in Relion, specifying eight asymmetric subunits for the reconstruction. Because of the irregular nature of the filaments, the optimal number of asymmetric units had to be experimentally tested by monitoring the quality of the final reconstruction and the FSC curve. The final helical symmetry parameters that were used for refinement were 21.2 Å for the rise and −85.8° for the twist. The final global resolution was estimated as 2.7 Å using FSC analysis with a fixed threshold at 0.143. The local resolution was calculated using Sparx/EMAN2 (73, 74) using previously described procedures (75). The 3D FSC (76) was obtained using the 3D FSC server (3dfsc.salk.edu), and the sampling compensation function (77) was calculated using the graphical user interface tool (78). Image analysis results are shown in Fig. S1 and summarized in Table S1.

### Atomic model refinement of $\mathrm{SgrA}I_{\mathrm{SP}/F}^{\mathrm{CA}}$ from cryo-EM

We used $SgrAI_{no-SP/F}^{MG}$, a model derived from a 3.5 Å cryo-EM map (PDB: 6OBJ (79)), to build and refine the model of $SgrAI_{SP/F}^{CA}$ into the new 2.7 Å cryo-EM map containing the SP. We performed an initial refinement within the Phenix (80) suite. Subsequently, model building/adjustment (including the SP and the addition of water molecules) and refinement were performed iteratively in Coot (81) and Phenix (62), and the statistics were examined using Molprobity (82) until no further improvements were observed. The final model was also evaluated using FSC analysis against the map and using EMRinger (83) to compare the fit of the model backbone into the cryo-EM map. The model statistics showed good geometry and matched the cryo-EM reconstruction (Fig. S1*C* and Table S1).

### Crystallization, X-ray diffraction data collection, structure solution, and refinement of DNA-free SgrAI ($\mathrm{SgrA}I_{\mathrm{apo}/D}^{\mathrm{CA}})$

Crystallization proceeded using the hanging-drop vapor diffusion method with the following conditions: crystallization well solution: 5% PEG 4000, 0.1 M imidazole (pH 6.5), 0.1 M NaCl, 5 mM Ca(OAc)_2_, drop: 1 μl of 6 mg/ml SgrAI dimer with threefold molar excess of PC-4 DNA in 10 mM Tris–HCl, 300 mM NaCl, 0.1 mM DTT, 0.1 mM EDTA, pH 8, and incubation at 17 °C. For data collection, crystals were harvested and exchanged into 10% PEG 4000, 0.1 M imidazole (pH 6.5), 0.3 M NaCl, 30% glycerol, and then flash frozen in liquid nitrogen. X-ray diffraction data collection proceeded at Stanford Synchrotron Radiation Lightsource BL 9-2 using Blu-Ice software (84). Data processing, including integration, scaling, and merging, was performed with XDS (85, 86) and SCALA (87, 88). Structure solution was performed using molecular replacement in PHASER (89) within the Phenix software suite (90) and searching for two copies of a single SgrAI chain from the prior structure 3DVO. The correct solution was ascertained by the formation of a dimer by the two placed single chains as well as by the final refinement statistics. Structure building and refinement proceeded through an interactive process using Coot (81, 91) and Phenix (90, 92, 93, 94).

### Structural comparisons—RMSD, alignments, DNA structure measurements, and protein–DNA interaction maps

RMSD calculations were performed using the University of California San Francisco (UCSF) Chimera package (95). The “Matchmaker” tool (with Needleman–Wunsch matrix for protein, and “nucleic” for DNA) was used with structure-based alignment using alpha carbons, phosphorus atoms, backbone atoms only, or all atoms of selected residues of selected chains. Analysis of subunit rotation was performed with UCSF Chimera, after first superimposing both chain A of 3DVO (18) and the cryo-EM model using Matchmaker in Chimera. The match command was used to calculate the rotation angle of chain B relative to each other. Structure figures were made with Pymol (96) or UCSF Chimera (95). DNA structural parameters, including base stacking, were calculated with 3DNA (97, 98). Protein–DNA interactions analyzed with DNAProDB (99, 100). Correlation coefficients between structures and maps were calculated with cryo-EM validations tools in Phenix (62).

## Data availability

The EM map and atomic model of $SgrAI_{SP/F}^{CA}$ have been deposited into the Electron Microscopy Data Bank and PDB under accession codes EMD-25404 and 7SS5, respectively. Coordinates and structure factor amplitudes for DNA-free nonfilamentous SgrAI ($SgrAI_{apo/D}^{CA}$) have been deposited into the PDB under accession code 7S8D.

## Supporting information

This article contains supporting information (78, 97, 98).

## Conflict of interest

The authors declare that they have no conflicts of interest with the contents of this article.
