# Supplementary material for: Pretransition state and apo structures of the filament-forming enzyme SgrAI elucidate mechanisms of activation and substrate specificity
Source: J Biol Chem. 2022 Feb 21;298(4):101760. doi: 10.1016/j.jbc.2022.101760 (PMC8960973; doi:10.1016/j.jbc.2022.101760)
Supplement: Supporting information Tables S1–S4 and Figures S1–S12 [file mmc1.docx]

**Supplementary Tables and Figures** for

“Pre-Transition State and Apo Structures of the Filament-Forming Enzyme SgrAI Elucidate Mechanisms of Activation and Substrate Specificity”,

Zelin Shan, Niloofar Ghadirian, Dmitry Lyumkis, N. C. Horton

**Contents:**

- **Table S1.** Cryo-EM data and structure refinement statistics
- **Table S2**. Data collection and refinement statistics of ${SgrAI}_{\frac{apo}{D}}^{CA}$
- **Table S3.** Base stacking areas of base pair steps in DNA bound to ${SgrAI}_{SP/F}^{CA}$and ${SgrAI}_{SP/D}^{CA}$
- **Table S4.** Protein-DNA interactions in ${SgrAI}_{SP/F}^{CA}$and ${SgrAI}_{SP/D}^{CA}$
- **Figure S1.** Cryo-EM data and validation.
- **Figure S2.** Examples of map quality at 1 σ contour level of ${SgrAI}_{SP/F}^{CA}$
- **Figure S3.** Active site arrangements in filamentous SgrAI structures with and without the SP
- **Figure S4**. Local base pair step parameters of DNA bound to ${SgrAI}_{SP/F}^{CA}$and ${SgrAI}_{SP/D}^{CA}$

## Figure S5. Overlays of sequence specific interactions between SgrAI and base pairs of the 8 bp recognition site in $\boldsymbol{SgrAI}_{\boldsymbol{SP/F}}^{\boldsymbol{CA}}$and $\boldsymbol{SgrAI}_{\boldsymbol{SP/D}}^{\boldsymbol{CA}}$

## Figure S6. Maps of SgrAI-DNA interactions in $\boldsymbol{SgrAI}_{\boldsymbol{SP/F}}^{\boldsymbol{CA}}$and $\boldsymbol{SgrAI}_{\boldsymbol{SP/D}}^{\boldsymbol{CA}}$

- **Figure S7**. RMSD between DNA-free SgrAI (${SgrAI}_{apo/D}^{CA})$ and DNA bound forms
- **Figure S8.** 2Fo-Fc map (contoured at 1σ) showing the ligation geometry around the site D Ca^2+^ in DNA-free SgrAI (${SgrAI}_{apo/D}^{CA})$
- **Figure S9**. Base stacking at the second base pair step in filamentous and non-filamentous SgrAI
- **Figure S10.** Overlay of the Ade2-Thy7 base pair in ${SgrAI}_{SP/F}^{CA}$ and ${SgrAI}_{SP/D}^{CA}$ and fit to CryoEM map of ${SgrAI}_{SP/F}^{CA}$
- **Figure S11.** Rotation of subunits leads to close approach of Gly284 to Cyt1
- **Figure S12.** Residues 284-287 shift closer to the first base pair of the recognition sequence

**Table S1. Cryo-EM Data and Structure Refinement Statistics**

| **EM Data Collection and Processing of** $\boldsymbol{SgrAI}_{\boldsymbol{SP/F}}^{\boldsymbol{CA}}$ | |
| --- | --- |
| Microscope | FEI Titan Krios |
| Voltage (kV) | 300 |
| Camera | Gatan K2 Summit |
| Magnification | 165,000 |
| Nominal defocus range (μm) | 0.8-2.0 |
| Exposure time (s) | 6 |
| No. of frames | 75 |
| Frame rate (frames per second) | 12.5 |
| Total fluence (e-/A^2^) | 30.5 |
| Fluence rate (e-/pixel/s) | 3.5 |
| Pixel size (Å) | 0.83 |
| No. of movies | 1047 |
| Total extracted particles | 278,865 |
| No. of particles in final map | 220,067 |
| Symmetry | Helical |
| Helical parameters (rise, Å / twist, °) | 21.2 / -85.8 |
| Resolution, Fourier shell correlation 0.143 (Å) | 2.7 |
| Local resolution range (Å) | 2.5-3.5 |
| Directional resolution range from 3D FSC (Å) | 2.5-3.1 |
| Sampling Compensation Factor (SCF) | 0.76 |
| Map-sharpening B factor (Å^2^) | -107.7 |
|  |  |
| **Atomic model refinement of** $\boldsymbol{SgrAI}_{\boldsymbol{SP/F}}^{\boldsymbol{CA}}$ |  |
| Model resolution (Å)  FSC ½ threshold | 2.7 |
| Model composition  Non-hydrogen atoms  Protein residues  Nucleotides  Waters  Ligands (Ca^2+^) | 6954 676 48 620 6 |
| R.m.s. deviations  Bond lengths (Å)  Bond angles (°) | 0.003 0.623 |
| Validation  MolProbity score  Clashscore  Poor rotamers (%) | 3.5 1.70 9.73 |
| Ramachandran plot  Favored (%)  Allowed (%)  Disallowed (%) | 0.83 96.88 3.12 0.00 |
| CaBLAM outliers (%) | 1.81 |
| Cis proline (%)  Twisted proline (%) Cβ outliers (%) | 0.00 0.00 0.00 |
| Average B-factors (min/max/mean, Å^2^)  Protein  Nucleotide  Ligand  Water | 0.00/55.19/24.40 8.12/88.94/39.15 18.77/33.40/27.39 8.16/42.56/21.97 |

## Table S2. Data collection and refinement statistics of $\boldsymbol{SgrAI}_{\boldsymbol{apo/D}}^{\boldsymbol{CA}}$

| Resolution range | 37.41Å - 2.025Å (2.097Å - 2.025Å) |
| --- | --- |
| Space group | P2_1_ |
| Unit cell | 70.11Å, 72.379Å, 78.64Å, 90°, 107.949°, 90° |
| Total reflections | 95022 (8680) |
| Unique reflections | 47684 (4428) |
| Multiplicity | 2.0 (2.0) |
| Completeness (%) | 97.50 (91.34) |
| Mean I/sigma(I) | 8.92 (2.16) |
| Wilson B-factor (Å^2^) | 22.11 |
| R-merge | 0.07552 (0.3917) |
| R-meas | 0.1068 (0.5539) |
| CC_1/2_ | 0.993 (0.522) |
| CC* | 0.998 (0.828) |
| Reflections used in refinement | 47674 (4428) |
| Reflections used for R-free | 1999 (186) |
| R-work | 0.2016 (0.2791) |
| R-free | 0.2458 (0.3300) |
| Number of non-hydrogen atoms | 5471 |
| macromolecules | 4908 |
| ligands | 2 |
| solvent | 561 |
| Protein residues | 643 |
| RMS(bonds) (Å) | 0.001 |
| RMS(angles) (°) | 0.43 |
| Ramachandran favored (%) | 98.41 |
| Ramachandran allowed (%) | 1.59 |
| Ramachandran outliers (%) | 0.00 |
| Rotamer outliers (%) | 0.41 |
| Clashscore | 1.14 |
| Average B-factor (Å^2^) | 25.61 |
| macromolecules | 24.89 |
| ligands | 28.82 |
| solvent | 31.82 |

Statistics for the highest-resolution shell are shown in parentheses.

## Table S3. Base Stacking Areas Areas of Base Pair Steps in DNA bound to $\boldsymbol{SgrAI}_{\boldsymbol{SP/F}}^{\boldsymbol{CA}}$and $\boldsymbol{SgrAI}_{\boldsymbol{SP/D}}^{\boldsymbol{CA}}$

| Stacked Bases  (5′->3′) | Atoms  Used | $\boldsymbol{SgrAI}_{\boldsymbol{SP/F}}^{\boldsymbol{CA}}$  (Current structure) (Å^2^) | $\boldsymbol{SgrAI}_{\boldsymbol{SP}\boldsymbol{/}\boldsymbol{D}}^{\boldsymbol{CA}}$  (3DVO)  (Å^2^) | Difference  (Å^2^) | Relative Change  (%) |
| --- | --- | --- | --- | --- | --- |
| 1^st^ Base Step  CACCGGTG  GTGGCCAC | | | | | |
| CA | **All** | 6.18 | 5.57 | 0.61 | 11 |
|  | **Ring** | 1.88 | 1.19 | 0.69 | 58 |
| TG | **All** | 0.24 | 0.48 | -0.24 | 50 |
|  | **Ring** | 0 | 0 | 0 | 0 |
| 2^nd^ Base Step  CACCGGTG  GTGGCCAC | | | | | |
| AC | **All** | 2.73 | 1.57 | 1.16 | 74 |
|  | **Ring** | **1.2** | **0.33** | **0.87** | **264** |
| GT | **All** | 8.7 | 8.36 | 0.34 | 4 |
|  | **Ring** | 4.53 | 3.85 | 0.68 | 18 |
| 3^rd^ Base Step  CACCGGTG  GTGGCCAC | | | | | |
| CC | **All** | 6.22 | 6.16 | 0.06 | 1 |
|  | **Ring** | 1.57 | 1.51 | 0.06 | 4 |
| GG | **All** | 3.65 | 4.49 | -0.84 | **-19** |
|  | **Ring** | 0.89 | 1.57 | -0.68 | **-43** |
| 4^th^ Base Step  CACCGGTG  GTGGCCAC | | | | | |
| CG | **All** | 2.46 | 2.76 | -0.3 | -11 |
|  | **Ring** | 0 | 0.03 | -0.03 | N/A |

## Table S4. Protein-DNA interactions in $\boldsymbol{SgrAI}_{\boldsymbol{SP/F}}^{\boldsymbol{CA}}$and $\boldsymbol{SgrAI}_{\boldsymbol{SP/D}}^{\boldsymbol{CA}}$^*^

| **Prot. Chain ID** | **Protein-DNA Interactions** | **Weak Protein-DNA Interactions** | **Total Buried Accessible Surface Area (Å^2^)** | **Total Hydrogen Bonds**  **(Hydrogen Bonds Made to Base Edges)** | **Total Number of van der Waals Interactions** |
| --- | --- | --- | --- | --- | --- |
| Per Half-Site (*i.e.* one SgrAI chain to one strand of DNA) | | | | | |
| $\boldsymbol{SgrAI}_{\boldsymbol{SP/F}}^{\boldsymbol{CA}}$ | | | | | |
| A | 66 | 5 | 1624.3 | 27 (11) | 149 |
| $\boldsymbol{SgrAI}_{\boldsymbol{SP/D}}^{\boldsymbol{CA}}$ | | | | | |
| A | 63 | 6 | 1400.7 | 30 (11) | 163 |
| B | 65 | 7 | 1452.3 | 28 (11) | 161 |
| **Only to the Outer Two Base Pairs, (*i.e.* Cyt1-Gua8’ and Ade2-Thy7’)** | | | | | |
| $\boldsymbol{SgrAI}_{\boldsymbol{SP/F}}^{\boldsymbol{CA}}$ | | | | | |
| A | 8 | 0 | 259.8 | 2 (0) | 9 |
| B | 22 | 0 | 733.5 | 11 (2) | 55 |
| $\boldsymbol{SgrAI}_{\boldsymbol{SP/D}}^{\boldsymbol{CA}}$ | | | | | |
| A | 5 | 0 | 211.8 | 1 (0) | 10 |
| B | 22 | 3 | 696.5 | 12 (2) | 66 |

^*^The structure of ${SgrAI}_{SP/F}^{CA}$is two-fold averaged, and therefore the statistics for only a single half-site are provided. However, the structure of ${SgrAI}_{SP/D}^{CA}$ derives from a crystal form where each half-site is independent, and therefore small differences are found between them.


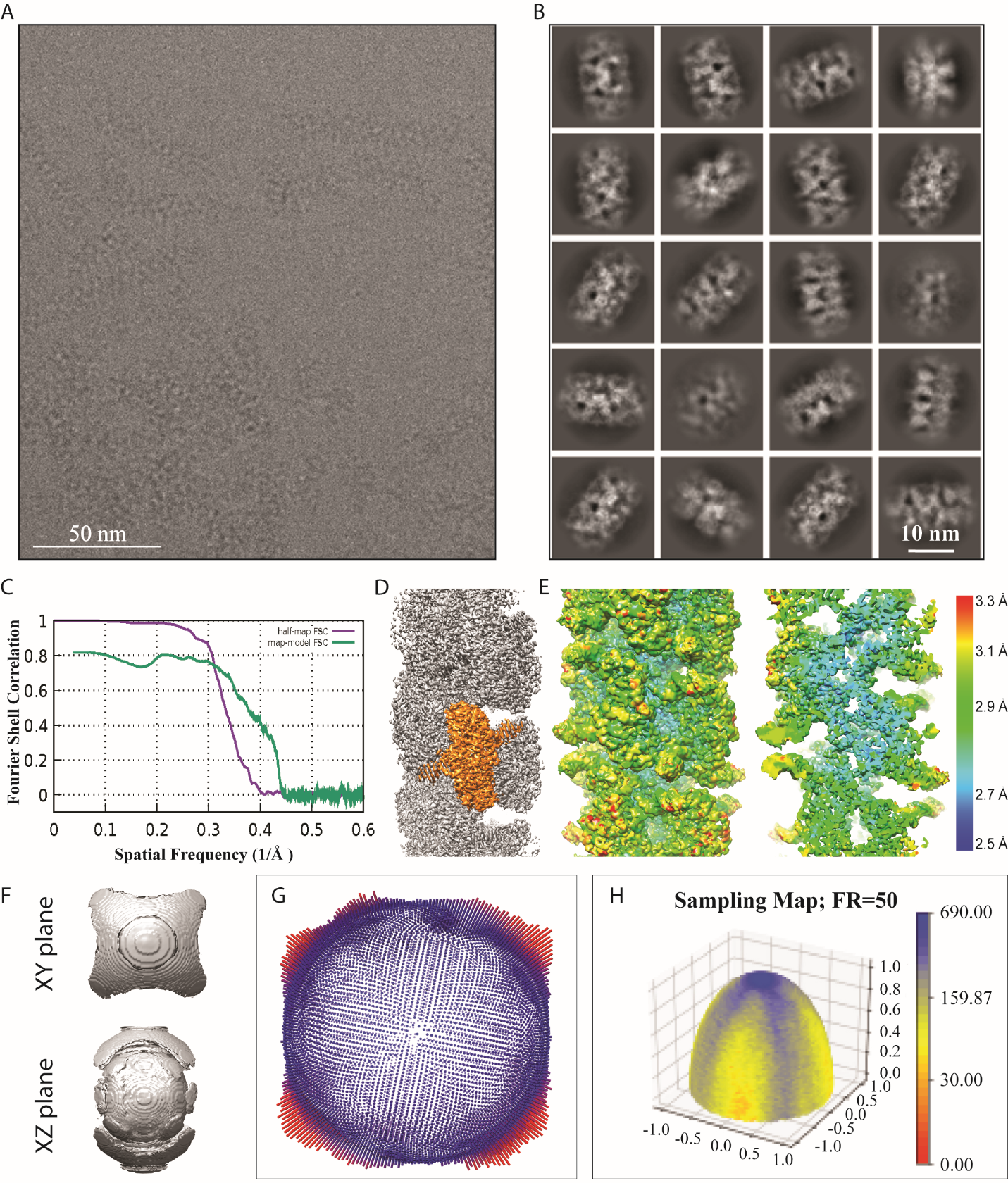


**Figure S1**: **Cryo-EM data and validation**. **A**. Cryo-EM micrograph of ${SgrAI}_{SP/F}^{CA}$. **B**. 2D class averages from particles selected from the data. **C**. Fourier Shell Correlation (FSC) curves showing half-map resolution of the map and map-to-model resolution of the model. **D**. Reconstruction of the filamentous assembly, with a single DBD segmented and highlighted in orange. **E**. Reconstruction from D colored by local resolution. **F**. 3D FSC isosurface displayed at a threshold of 0.5 with two perpendicular planar views. **G**. Euler plot corresponding to the particles used in the cryo-EM reconstruction. **H**. Surface sampling plot(1) corresponding to the Euler angle distribution, calculated with a Fourier radius set to 50 voxels.


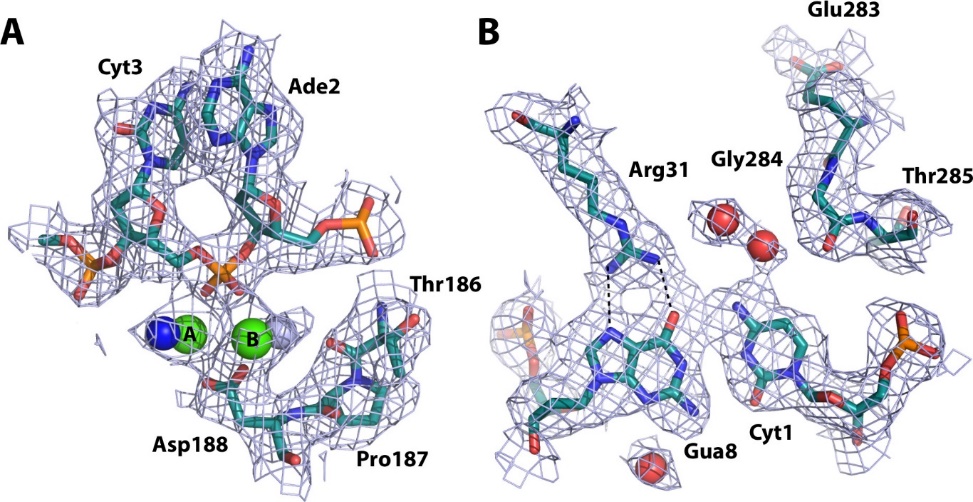


**Figure S2.** **Examples of map quality at 1 σ contour level of** $\boldsymbol{SgrAI}_{\boldsymbol{SP/F}}^{\boldsymbol{CA}}$. **A**. Selected atoms in and around the active site. Solvent shown in light and dark blue spheres, Ca^2+^ ions in green spheres. **B.** Selected atoms around the first base pair of the recognition sequence and Arg31 of the Arg31 loop. Solvent water molecules shown as red spheres. Hydrogen bonds between Arg31 and Gua8 shown as dashed lines.


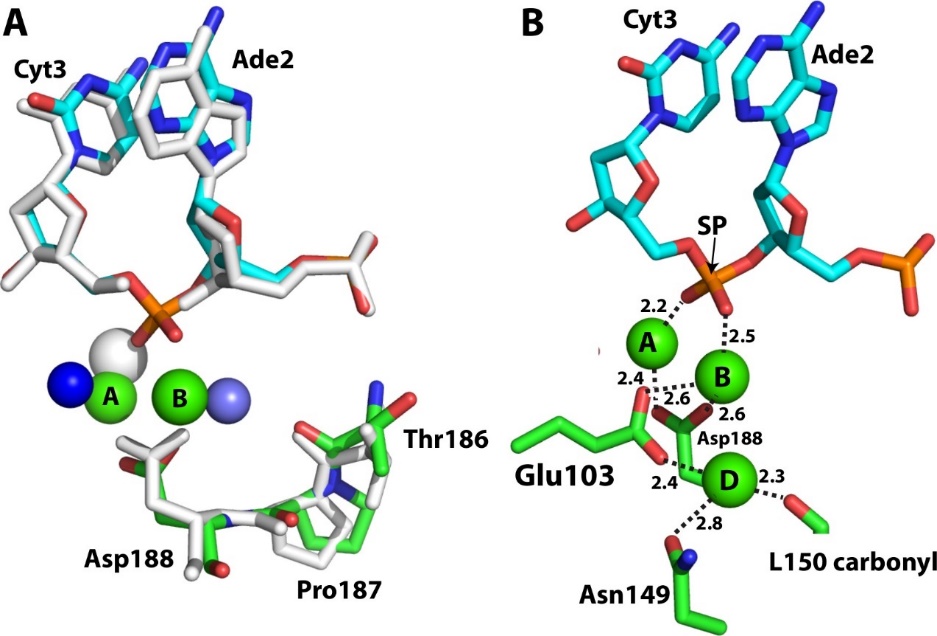


**Figure S3.** **Active site arrangements in filamentous SgrAI structures with and without the SP. A.** Superposition of ${SgrAI}_{SP/F}^{CA}$ (carbon in cyan) containing the SP with the prior structure of filamentous SgrAI bound to primary site DNA but lacking the SP (${SgrAI}_{no-SP/F}^{MG}$**,** white). ${SgrAI}_{no-SP/F}^{MG}$ contains one Mg^2+^ ion in site A (white sphere), while ${SgrAI}_{SP/F}^{CA}$contains two Ca^2+^ ions (green spheres)(in sites A and B). Site D Ca^2+^ is outside the field of view in this orientation. The blue spheres are Ca^2+^ ligated water molecules in ${SgrAI}_{SP/F}^{CA}$, the water in dark blue is in an orientation for in-line attack on the SP phosphorus atom, and the water in light blue ligates the site B Ca^2+^ and hydrogen bonds to the Thr186 carbonyl oxygen atom. **B**. Active site of chain B of ${SgrAI}_{SP/F}^{CA}$ showing Ca^2+^ ions in sites A, B, and D (green spheres). SP indicates scissile phosphate. Distances are given in Å.


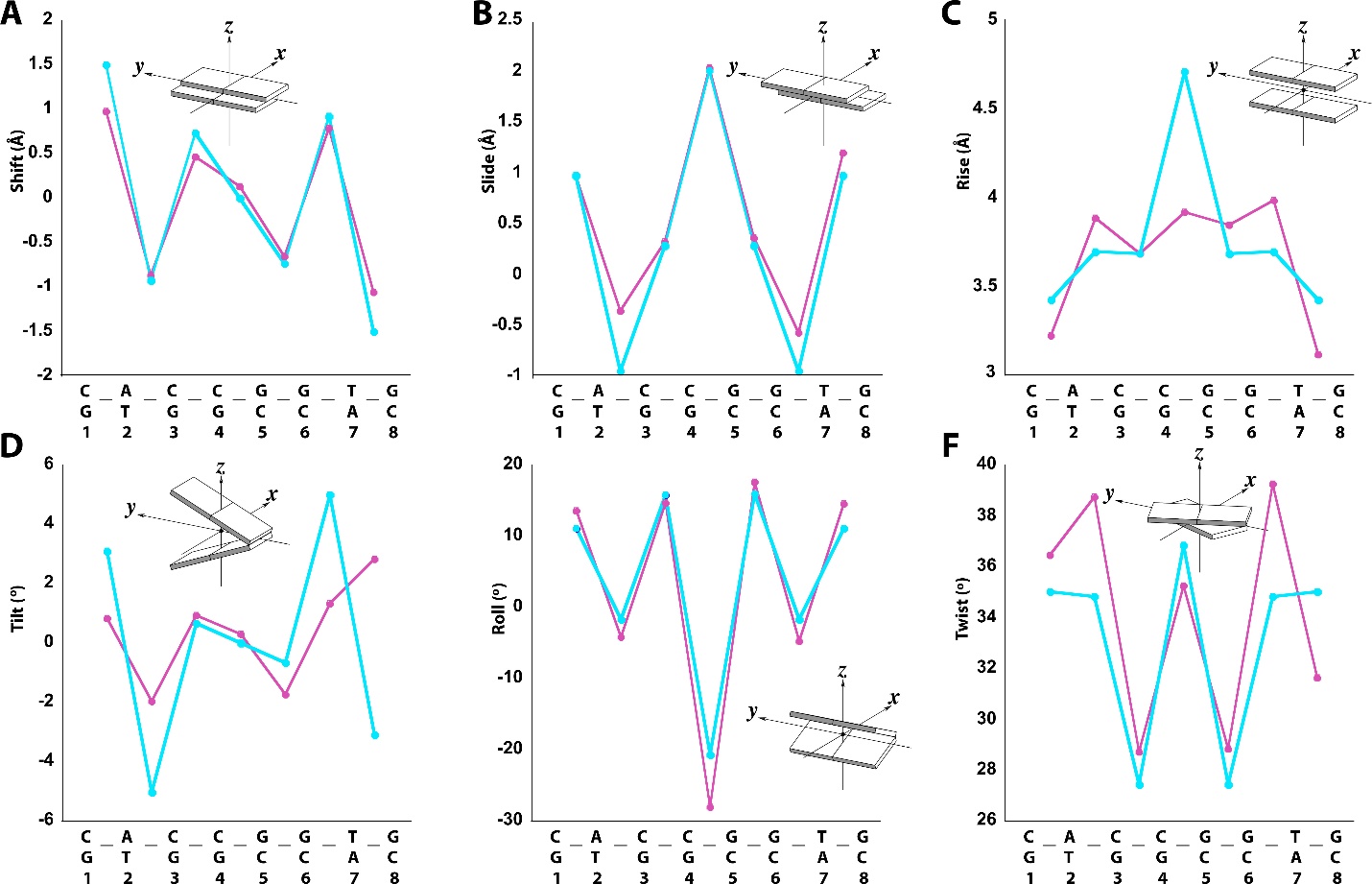


**Figure S4. Local Base Pair Step Parameters. A,** Shift, **B**, slide, **C**, rise, **D**, tilt, **E**, roll, and **F**, twist calculated for the 8 base pair recognition sequence bound to filamentous SgrAI (${SgrAI}_{SP/F}^{CA}$, light blue) and non-filamentous, dimeric SgrAI (${SgrAI}_{SP/D}^{CA}$, magenta). Inset diagrams in each panel illustrate measured parameter (helical axis along z, base pairs in the xy plane, gray shaded side indicates minor groove edge). Calculations performed with 3DNA(2,3).


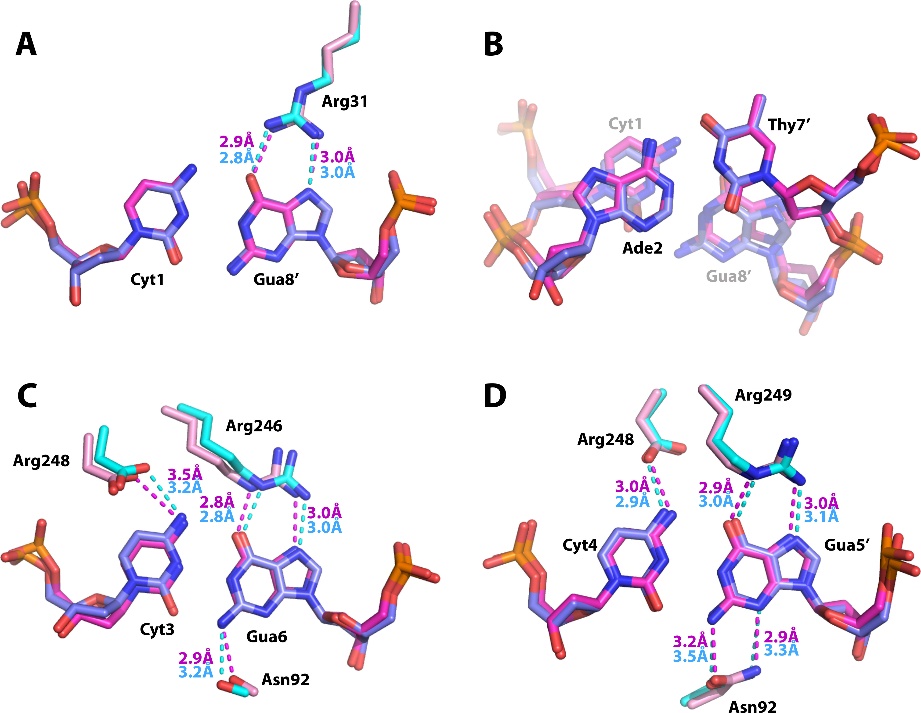


**Figure S5. Overlays of sequence-specific interactions between SgrAI and base pairs of the 8 bp recognition site in** $\boldsymbol{SgrAI}_{\boldsymbol{SP/F}}^{\boldsymbol{CA}}$**and** $\boldsymbol{SgrAI}_{\boldsymbol{SP/D}}^{\boldsymbol{CA}}\boldsymbol{.}$**A.** Sequence specific interactions between SgrAI and bound DNA at the first bp of the 8 bp recognition sequence. Atoms colored by type with carbon atoms of filamentous SgrAI (${SgrAI}_{SP/F}^{CA}$) in light and dark blue, and non-filamentous SgrAI (${SgrAI}_{SP/D}^{CA})$in light and dark pink. Base atoms were used in the superposition. **B.** As in A, at the second bp of the 8 bp recognition sequence. No sequence specific contacts are made to the second (and seventh) base pair. Instead, a large distortion in the form of unstacking of the Thy7 from the Gua8 base is seen, and is preserved in both structures. **C.** As in A, but at the third base pair. **D.** As in A, but at the fourth base pair.


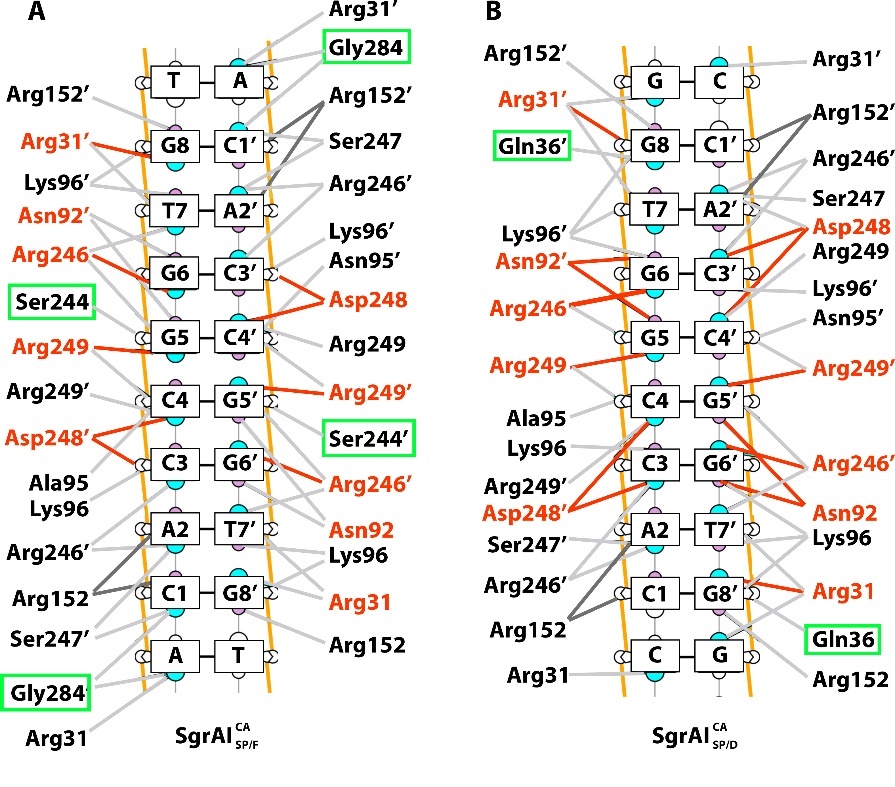


## Figure S6. Maps of SgrAI-DNA interactions in $\boldsymbol{SgrAI}_{\boldsymbol{SP/F}}^{\boldsymbol{CA}}$and $\boldsymbol{SgrAI}_{\boldsymbol{SP/D}}^{\boldsymbol{CA}}$. A. Filamentous SgrAI bound to DNA ($\boldsymbol{SgrAI}_{\boldsymbol{SP/F}}^{\boldsymbol{CA}}$). B. Non-filamentous SgrAI bound to DNA ($\boldsymbol{SgrAI}_{\boldsymbol{SP}\boldsymbol{/}\boldsymbol{D}}^{\boldsymbol{CA}}$, PDB code 3DVO). In both panels A-B, contacts resulting from either van der Waals interactions or those that lead to buried solvent accessible surface area are shown as light grey lines, and hydrogen bonding interactions are shown as either red or dark grey lines. Red lines and red text indicate sequence-specific hydrogen bonding interactions to the DNA. Residues in green boxes indicate contacts that are unique to each structure. Light blue and light pink semi-circles indicate major and minor groove atoms of each base.


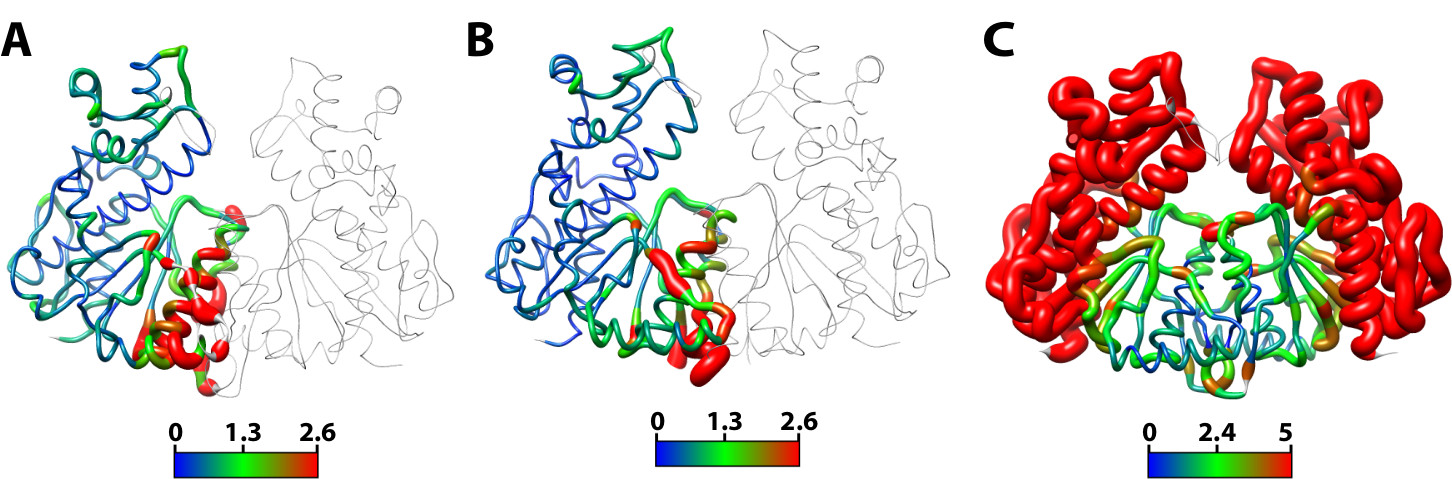


**Figure S7. RMSD between DNA-free SgrAI (**$\boldsymbol{SgrAI}_{\boldsymbol{apo/D}}^{\boldsymbol{CA}}\boldsymbol{)}$ **and DNA bound forms. A.** Ribbon diagram of filamentous, DNA bound SgrAI (${SgrAI}_{SP/F}^{CA}$) colored and with ribbon thickness according to the RMSD when compared to DNA-free SgrAI (${SgrAI}_{apo/D}^{CA}$), aligned on a single chain. The chain that is not used in the calculation is shown in grey. Color key for RMSD values given in Å. **B.** As in B, but showing the RMSD values between the non-filamentous DNA bound form (PDB code 3DVO, ${SgrAI}_{SP/D}^{CA}$) and ${SgrAI}_{apo/D}^{CA}$, mapped onto ${SgrAI}_{SP/D}^{CA}$. **C.** As in A but using both chains in the superposition.


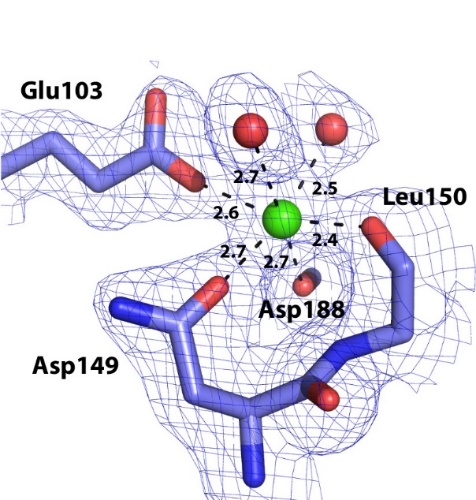


**Figure S8. 2Fo-Fc map (contoured at 1σ) showing the ligation geometry around the site D Ca^2+^ in DNA-free SgrAI (**$\boldsymbol{SgrAI}_{\boldsymbol{apo/D}}^{\boldsymbol{CA}}\boldsymbol{)}$**.** Distances are in Å and map shown at 1σ. Ca^2+^ shown as green sphere, and ligated water molecules as red spheres.

**
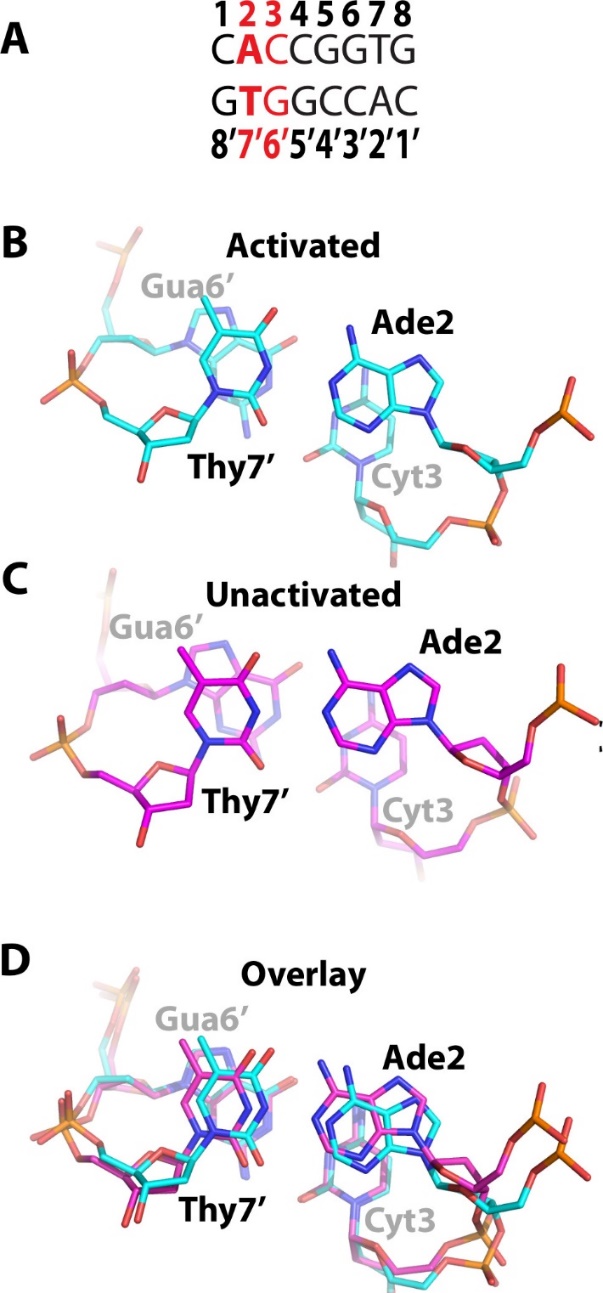
**

**Figure S9.** **Base stacking at the second base pair step in filamentous and non-filamentous SgrAI.** **A**. Sequence and numbering of primary site DNA bound to SgrAI in the different structures. **B.** Second and third base pair of the SgrAI primary site recognition sequence, showing overlap of bases in ${SgrAI}_{SP/F}^{CA}$. View is from Ade2:Thy7′ above Cyt3:Gua6′. **C.** As in A, but the non-filamentous ${SgrAI}_{SP/D}^{CA}$, low activity structure of SgrAI with primary site DNA (PDB code 3DVO). **D.** Overlay of structures shown in B and C using all base atoms of Cyt3 and Gua6′ to emphasize shift in positioning of Ade2:Thy7′.


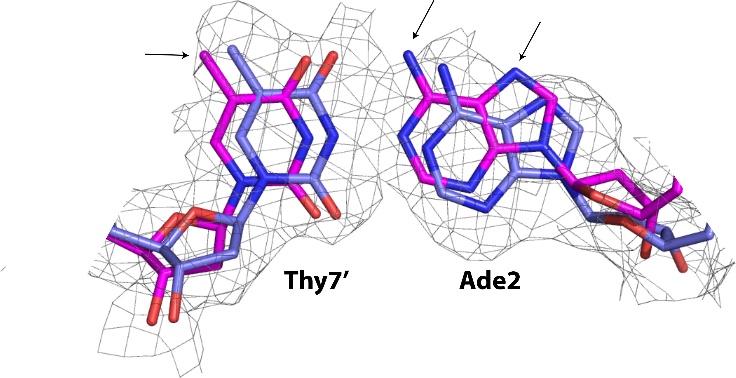


**Figure S10.** **Overlay of the Ade2-Thy7’ base pair in** $\boldsymbol{SgrAI}_{\boldsymbol{SP/F}}^{\boldsymbol{CA}}$ **and** $\boldsymbol{SgrAI}_{\boldsymbol{SP/D}}^{\boldsymbol{CA}}$ **and fit to Cryo-EM map of** $\boldsymbol{SgrAI}_{\boldsymbol{SP/F}}^{\boldsymbol{CA}}$**.** Base atoms of Cyt1-Gua8′ were used to superimpose the two structures to view the shifted position of Ade2-Thy7′ between the two structures. Cryo-EM map shown at 1 σ in blue. ${SgrAI}_{SP/F}^{CA}$ shown with blue carbon atoms, ${SgrAI}_{SP/D}^{CA}$shown with magenta carbon atoms. Arrows identify regions where the ${SgrAI}_{SP/D}^{CA}$coordinates fall outside of the map.

**
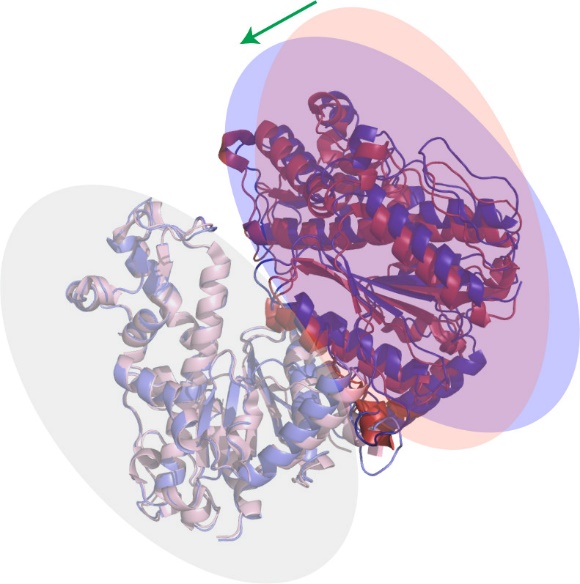
**

**Figure S11. Rotation of subunits leads to close approach of Gly284 to Cyt1.** SgrAI dimer from filamentous ${SgrAI}_{SP/F}^{CA}$(dark and light blue), and dimeric ${SgrAI}_{SP/D}^{CA}$ (red and pink), with subunits shown in lighter and darker colors. The rotation of 11° degrees leads to the close approach of Gly284 to Cyt1 (see **Fig. 7**).

**
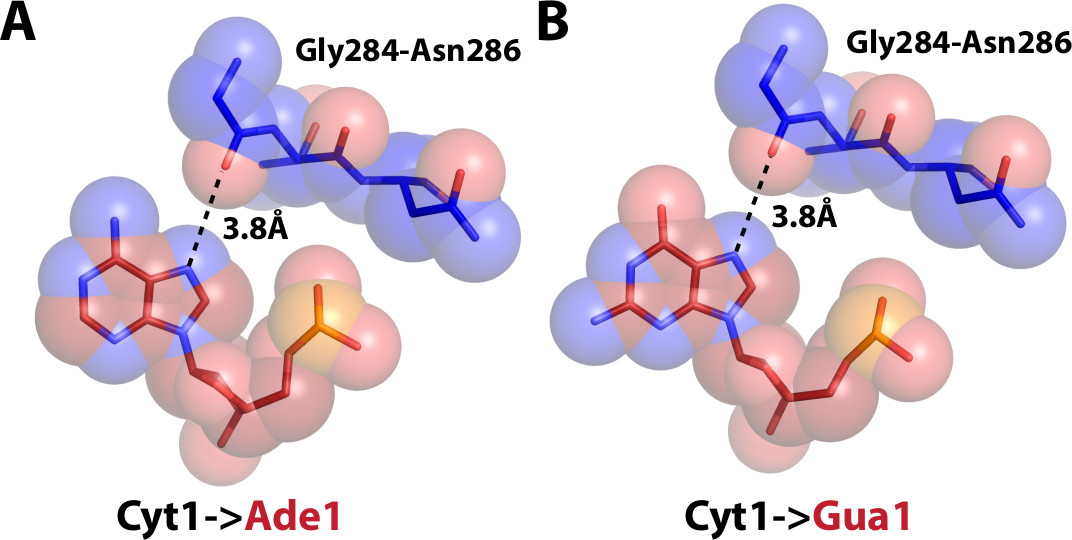
**

**Figure S12. Residues 284-287 shift closer to the first base pair of the recognition sequence. A.** Superposition of an Ade base in place of Cyt1 of ${SgrAI}_{SP/F}^{CA}$ showing the close approach of the carbonyl oxygen of Gly284 to the N7 base atom. **B.** As in A with superposition of an Ade base in place of Cyt1.

**References**

1. Baldwin, P. R., and Lyumkis, D. (2021) Tools for Visualizing and Analyzing Fourier Space Sampling in Cryo-Em. *Prog Biophys Mol Biol* **160**, 53-65

2. Lu, X. J., and Olson, W. K. (2003) 3dna: A Software Package for the Analysis, Rebuilding and Visualization of Three-Dimensional Nucleic Acid Structures. *Nucleic Acids Res* **31**, 5108-5121

3. Lu, X. J., and Olson, W. K. (2008) 3dna: A Versatile, Integrated Software System for the Analysis, Rebuilding and Visualization of Three-Dimensional Nucleic-Acid Structures. *Nat Protoc* **3**, 1213-1227
